# Amide naphthotube as a novel supramolecular sequestration agent for tetracaine and decamethonium

**DOI:** 10.7150/thno.93654

**Published:** 2024-08-19

**Authors:** Cheng-Da Zhao, Wei Cai, Wen-Jie Chen, Huan Yao, Song-Meng Wang, Kailin Li, Yan-Long Ma, Li-Li Wang, Liu-Pan Yang

**Affiliations:** 1 The Affiliated Nanhua Hospital, School of Pharmaceutical Science and School of Basic Medical Sciences, Hengyang Medical School, University of South China, Hengyang, 421001, China.; 2 School of Pharmaceutical Sciences, Hunan University of Medicine, Huaihua, 418000, China.; 3 Department of Chemistry, Southern University of Science and Technology, Xueyuan Blvd 1088, Shenzhen, 518055, China.; 4 School of Chemistry and Chemical Engineering and Guangdong Cosmetics Engineering & Technology Research Center, Guangdong Pharmaceutical University, Zhongshan 528458, China.

**Keywords:** supramolecular sequestration, detoxification, tetracaine, decamethonium, amide naphthotube

## Abstract

Rationale: Anesthetics are widely used for optimizing surgical conditions, postoperative pain management, and treating various chronic pain conditions. Tetracaine and decamethonium are representative drugs of local anesthetics and neuromuscular blocking agents, respectively. However, overdose and toxicity of the drugs always lead to serious adverse events. Thus, there is a strong demand for effective antidotes.

Methods: The binding interactions of amide naphthotubes with tetracaine and decamethonium were systematically studied using ^1^H NMR, ITC, and DFT calculations. The antidotal effects of amide naphthotube to tetracaine toxicity were assessed in vitro and in vivo, and the mechanism of detoxification was explored at a cellular level. Additionally, mouse models were established to evaluate the reversal activities of amide naphthotube on decamethonium-induced mortality and muscle relaxation, and the reversal mechanism was investigated through pharmacokinetic experiments.

Results: We have demonstrated that the anti-isomer of amide naphthotube exhibits significant binding affinities towards tetracaine (*K*_a_ = 1.89×10^7^ M^-1^) and decamethonium (*K*_a_ = 1.01×10^7^ M^-1^) in water. The host displayed good biocompatibility both in vitro and in vivo. The administration of amide naphthotube following tetracaine overdose in mouse models notably increased the overall survival rate, indicating its effective antidotal properties. The host could reverse the tetracaine-induced Na^+^ channels blockage at the cellular level. Moreover, the injection of amide naphthotube also reversed the mortality and paralysis induced by decamethonium in mouse models following a pharmacokinetic mechanism.

Conclusion: An emerging artificial receptor, amide naphthotube, has strong binding affinities towards tetracaine and decamethonium. It functions as a supramolecular antidote for tetracaine poisoning and a reversal agent for decamethonium by selectively sequestering these compounds in vivo.

## Introduction

Medications are crucial tools in managing and conquering diseases, contributing to the increased lifespan and improved quality of life seen over the past century. However, medications also carry potential risks, as their toxic side effects can persist even after their beneficial effects have ceased. Moreover, the accumulation of exogenous environmental toxins and endogenous toxic metabolites can result in significant organ damage and threaten human health 1. To address medication overdose and toxicity, pharmacodynamic and pharmacokinetic methods are utilized. Pharmacodynamic methods involve the competitive binding of small molecule antagonists to block and disrupt medication actions 2. In contrast, pharmacokinetic approach methods seek to lower medication concentrations, deactivate their biological activity, and facilitate their elimination from the body 3, 4. Comparatively, the latter approach does not necessitate an exact comprehension of the underlying mechanisms of drug action, and notable advancements have been achieved utilizing this methodology.

Since 2000, there has been rapid development in the field of aqueous molecular recognition based on synthetic hosts 5, laying a solid foundation for applications in the pharmaceutical industry 6, 7, 8, 9, 10, 11, 12, 13, 14, 15. In this area, synthetic supramolecular sequestration agents are highly representative due to their easy modification, high thermal and chemical stability, and abiotic nature 16, 17, 18, 19, 20. For instance, Sugammadex, a derivative of γ-cyclodextrin, can effectively antagonize muscle relaxants rocuronium and vecuronium by forming tight complexes and has achieved impressive commercial success with annual sales revenue exceeding $1 billion 21. Besides, the application of other artificial hosts such as calixarenes 22, (acyclic)cucurbiturils 23, 24, 25, pillararenes 26, 27, 28 and biphen[n]arene 29 in this field have also been explored. The guests include neuromuscular blockers, anesthetics, toxic substances, and drugs of abuse 16-20. Existing supramolecular sequestration agents are typically derived from modifying classic macrocyclic hosts. Developing new host systems to sequester complex or polar guests that are resistant to physiological salt and serum proteins is highly desirable.

**Figure 1:**
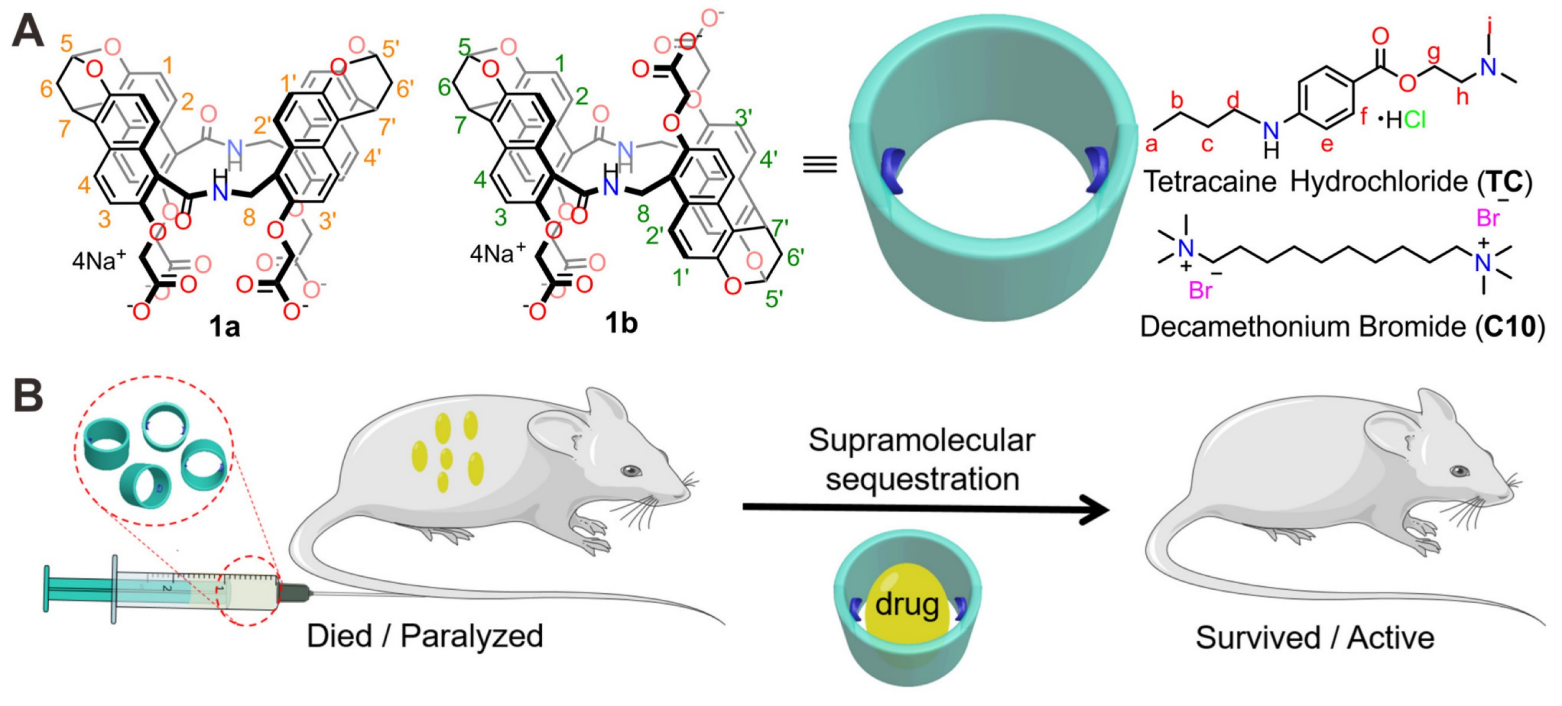
** Drug reversal by amide naphthotube. (A)** Chemical structures of amide naphthotube and drug molecules. **(B)** Cartoon depicting supramolecular sequestration of **TC** and **C10** in vivo.

Local anesthetics effectively and reversibly block sensory nerve impulses in specific areas, which is crucial in postoperative pain management and treatment of chronic pain conditions 30, 31, 32. Tetracaine (**TC**, Figure 1A) is a potent ester-type drug commonly used for various types of anesthesia 33. In recent years, with the emergence of the medical cosmetic industry, tetracaine has increasingly been applied in procedures such as cosmetic surgery, eyebrow tattooing, and tattooing 34. However, incidents of **TC** poisoning, shock, and even death have occurred due to improper administration leading to bloodstream entry, excessive dosage, or allergic reactions, posing significant threats to public health 35, 36, 37, 38. Still, there is no specific antidote for **TC** poisoning in clinical practice 39. In addition, neuromuscular blocking agents are commonly used in anesthesia to relax skeletal muscle movements and improve surgical conditions. Rapid recovery after surgery is essential for muscle function and to prevent lingering effects. Decamethonium (**C10**) is a conventional neuromuscular blocking agent with a lengthy record of clinical and experimental applications. However, the restricted metabolic clearance in the body and the tendency to cause muscle pain and other adverse reactions, combined with the lack of a suitable antidote for clinical management, significantly limit the clinical use of **C10** 40, 41, 42, 43. Therefore, it is clinically significant to develop an effective antidote that can alleviate or reverse the toxicity of **TC** and **C10**.

In recent years, amide naphthotubes (Figure 1A, **1a and 1b**) have garnered significant attention in supramolecular chemistry due to their unique recognition capabilities in water 44. Initially reported by Glass in 2004 for the selective sensing of lipids 45, our group has comprehensively studied the guest binding scope, recognition mechanism, physico-organic characteristics, and functional applications of amide naphthotubes 46, 47, 48. Amide naphthotubes have the ideal properties for in vivo sequestration applications: Firstly, they have low cytotoxicity and have realized some biomedical applications 49, 50; Secondly, carboxylate-substituted hosts are highly water-soluble and do not aggregate at high concentrations (350 mM) 50; Thirdly, the hosts have deep hydrophobic cavities with buried hydrogen-bonding sites, allowing for selective recognition of hydrophilic and polar organic molecules, which is quite complementary to traditional macrocyclic receptors; Finally, their binding affinities to neutral organic molecules in aqueous environments generally unaffected by the presence of small biomolecules and inorganic salts 51. Herein, we investigated and demonstrated that amide naphthotube could bind **TC** and **C10** with binding affinities more than 10^7^ M^-1^ in water, and exhibited good biocompatibility in mouse models. Moreover, the synthetic receptor could serve as a sequestration agent for **TC** and **C10** via supramolecular pharmacokinetic approaches to reverse the toxicity of **TC** and the neuromuscular blocking effect of **C10** (Figure 1B). More importantly, it may serve to meet the longstanding medical requirements of the healthcare community.

## Methods

### General methods

The reagents and guest molecules used in this study were commercially available and used without further purification unless stated otherwise. Ultrapure water was obtained from the Chuangchun pure water machine CCH-H200. ^1^H NMR spectra were recorded using a Bruker Avance 500 NMR spectrometer, and chemical shifts are reported in ppm with residual solvents or sodium methanesulfonate as internal standards. Amide naphthotubes **1a** and **1b** (Chemical Formula: C_58_H_38_N_2_Na_4_O_18_, Molecular Weight: 1142.8971 g/mol) were synthesized as described previously 52.

### ITC experiments

ITC experiments were conducted in water or fetal bovine serum (FBS) at a certain temperature using a Malvern MicroCal PEAQ-ITC Automated instrument. Typically, 280 µL of the host solution in water or 100% FBS was placed in the sample cell, while 38 µL of the guest solution was loaded into the injection syringe. The titration consisted of an initial 0.4 µL injection followed by 18 consecutive 2 µL injections with a 120 s interval between injections. All solutions were degassed before titration. The data was analyzed using the instrument's internal software and fit to a "one set of binding sites" model.

### Computational Methods

The quantum chemistry calculations were conducted using the Gaussian 16 package to optimize the structures of host-guest complexes. This was achieved through density functional theory (DFT) with calculations (ωB97xd/(ma)-def2-SVP for energy-minimizing, ωB97xd/ma-def2-TZVPP for single point energy) in water (PCM/SMD model) at 298 K 53, 54. Additionally, an independent gradient model based on Hirshfeld partition (IGMH) analysis was carried out using the Multiwfn 3.8 (dev) program, and the molecular plots were visualized with the VMD 1.9.3 program 55, 56.

### Hemolysis test

Fresh blood was collected from Sprague-Dawley rats from the orbital sinuses. The red blood cells (RBCs) were extracted by centrifugation and diluted to 2% (v/v) with saline. Various concentrations of **1b** ranging from 12.5 to 800 μM were added to the 2% RBCs. Ultrapure water and saline served as the positive and negative controls, respectively. Following a 3-hour incubation at 37°C, the supernatants were obtained through centrifugation. The hemolysis rate was determined by measuring the A450 nm of the supernatants from different samples using the following equation:

Hemolysis ratio (%) = (OD_sample_ - OD_negative control_)/(OD_positive control_ - OD_negative control_) × 100%

### Toxicity evaluation of 1b with *i.v.* administration

Lethal **1b** poisoning mouse model: Female Balb/c mice aged 8-10 weeks were randomly assigned to four groups (n = 8 for each group). The mice were *i.v.* injected with **1b** saline solutions at 500, 750, and 1000 mg/kg. In the control group, mice received the same amount of saline. Their weight was measured every two days, and their behavior was observed daily for signs of illness. After 21 days, the animals were sacrificed, and major organs such as the heart, liver, kidneys, lungs, and spleen were collected and weighed to calculate the organ index.

Potential toxicity evaluation of **1b**: Female Balb/c mice (8-10 weeks old) weighing 20-25 g were randomly divided into groups (n = 15 for each group). **1b**/saline solutions (20 mg/mL) were prepared and filtered through a 0.22 µm membrane and then *i.v.* administered to mice at a dose of 100 mg/kg body weight. The control group received 50 µL saline/10 g body weight. Mice were monitored daily for weight changes and signs of illness. Major organs of three mice from each group were collected for histopathological analysis after 1, 3, 7, and 14 days. After 1 and 14 days, blood samples were collected for hematologic analysis (n = 6 for each group). The serum was separated for liver and renal function markers detection. The tissues were initially fixed in 4% paraformaldehyde, then submitted to Hunan AiFang Biological Co., Ltd., for paraffin embedding and sectioning. The sections were subsequently stained with H&E and examined using an optical microscope by the company. Whole blood cell (WBC), red blood cell (RBC), platelet (PLT), hemoglobin (HGB), alanine aminotransferase (ALT), aspartate aminotransferase (AST), creatinine (CRE) and urea were analyzed by Wuhan Servicebio Technology Co., Ltd.

### The evaluation of the capacity of 1b for reversing TC toxicity

Cells experiments: AC16 cells were cultured in DMEM/F12 with 10% FBS, 1% penicillin, and 1% streptomycin at 37 °C with 5% CO_2_, and passaged every 2 days. They were seeded into 96-well plates at a density of 5-8×10^3^ cells per well and cultured overnight. Then, different concentrations of **TC**, **1a**, **1b**, **TC**@**1a** and **TC**@**1b** mixture solution at various concentrations (25, 50, 100, 100, 200, 400, 800, and 1600 μM) were added to replace the culture medium for 24 h at 37 °C. After incubation, Cell Counting Kit-8 solution (10 μL per well) was added, and cells were incubated for 4 h at 37 °C. The optical density values at 450 nm were measured using a microplate reader to assess cell viability as a percentage of control cells. Finally, IC_50_ values were calculated using GraphPad Prism 8.0.2 software.

According to the IC_50_ value of **TC**, the concentration of **TC** was fixed at 400 μM, the cells were treated with a series of fresh medium containing different fixed molar ratios of **TC** at a minimum of 8 concentrations (1:0, 1:0.00625, 1:0.125, 1:0.25, 1:0.5, 1:1, 1:2 and 1:4) of **TC** and **1b** for 24 h at 37 °C. The assay and cell viability were calculated as in the cell experiment above.

Lethal **TC** poisoning mouse model: the lethal dose of *i.v.* administered **TC** was calculated in mice (8-10 week old female Balb/c mice, weighing 20-25 g) using Dixon's up-and-down method. The initial dose of **TC** given to the first mouse was 9.0 mg/kg, and the standard dose ratio of **TC** given to the adjacent mouse was 0.9. If the animal survives, the subsequent animal receives a higher dose. If the animal dies, the following animal receives a lower dose. This process was repeated until at least seven crossover points were achieved. Probit analysis was employed to determine the **LD50** (50% lethal dose) and **LD95** (95% lethal dose) of **TC**, along with their corresponding **CI** (confidence intervals). Sequence diagrams and dose-response curves were generated using GraphPad Prism 9 software.

Assessment of the antidotal effects of **1b** in a mouse model of lethal **TC** poisoning: Female Balb/c mice aged 8-10 weeks and weighing 20-25 g were randomly allocated to various groups (n = 8 for each group). Solutions of **1b**/saline at concentrations of 4, 8, 12, and 16 mg/mL were prepared. The mice were initially *i.v.* injected with **TC** at 9.0 mg/kg, followed by *i.v.* administration of **1b** at doses of 20, 40, 60, and 80 mg/kg. The control group received an *i.v.* injection of 100 µL of saline per 20 g. The mice were monitored for survival for 24 hours. Furthermore, the safety of mice with 100% survival was evaluated by recording their body weights and observing their behavior daily for any signs of illness.

Determination of the plasma membrane potential: AC16 cells were seeded into 12-well plates and cultured until reaching 80-90% confluence. Following removal of the culture medium, cells were exposed to HBSS (Hank's Balanced Salt Solution) containing 0, 100, 200, 400, 600, and 800 mM **TC** for 10 minutes at 37 °C. Subsequently, cells were collected and incubated with 200 nM Bis-(1,3-Dibutylbarbituric Acid)-Trimethine Oxanol (DiBAC4(3), Solarbio) for 20 minutes at 37 °C. The fluorescence intensity of cells was then assessed using flow cytometry. To determine the impact of **1b** (ammonium salt) on plasma membrane potential, 25×10^4^ AC16 cells were co-incubated with 400 µM **TC** and varying concentrations of **1b** (0, 400, 800, and 2000 µM) or exclusively with **1b** (2000 µM) in HBSS for 10 minutes at 37 °C. Plasma membrane potential was subsequently evaluated using the aforementioned methodology.

Determination of intracellular sodium levels: 3×10^5^ AC16 cells were seeded in 6-well plates with 2 mL of medium. After 12 hours, the cell medium was replaced with 400 µM **TC** and varying concentrations of compound **1b** (ammonium salt, 0, 400, 800, and 2000 µM) or 2000 µM **1b** in HBSS for 10 minutes at 37 °C. Control cells were treated with 2 mL HBSS for 10 minutes at 37 °C. After washing with PBS, cells were lysed in 300 µL of 1% Triton X-100 in saline with a 1% proteinase inhibitor cocktail from Meilunbio. The lysates were centrifuged at 12,000 ×g for 15 minutes, and the supernatant was collected for sodium level determination (7600 Modular-Hitachi-Chemistry Analyzer) and total protein content BCA assays (Epizyme Biotech). The sodium-to-protein ratio was calculated, presenting results as relative intracellular sodium content compared to the control group.

### The evaluation of the capacity of 1b for reversing C10 toxicity and muscle relaxation

Lethal **C10** poisoning mouse model: the lethal dose of *i.v.* administered **C10** was calculated using Dixon's up-and-down method similar to **TC.** The initial dose of **C10** was 0.9 mg/kg. Subsequent doses for each mouse were adjusted by ±0.1 mg/kg based on the outcome of the preceding animal.

Assessment of the antidotal effects of **1b** in a mouse model of lethal **C10** poisoning: Female Balb/c mice (8-10 weeks old) weighing 20-25 g were randomly divided into groups (n = 8 for each group). Solutions of **1b**/saline at concentrations of 0.4, 0.8, 1.2, and 1.6 mg/mL were prepared. The mice were first injected intravenously with **C10** at a dose of 0.9 mg/kg, followed by *i.v.* administration of **1b** at doses of 2, 4, 6, and 8 mg/kg. The control group was given a 100 µL saline *i.v.* injection per 20 g. The survival of the mice was monitored within 24 hours. To further assess the well-being of the mice with a 100% survival rate, their body weights were recorded and their behavior was observed daily for any signs of illness.

Determination of **C10**-induced paralysis dose in a mouse model: The movement behavior was conducted with the Rotarod rotor (ZB-200). Female Balb/c mice (8-9 weeks old) weighing 18-22 g were placed on the Rotarod rotor and trained three times for 30 minutes, three times a day for three consecutive days. On day 4, Dixon's up-and-down method was employed to explore the effective dose: The initial dose of **C10** to the first mouse was 0.6 mg/kg, and the standard dose ratio of **C10** given to the adjacent mouse was 0.8. If the former mouse had a response to injection (''response'' defined as the mice could not move on the rotor for 30 seconds after 30 seconds of **C10** injection), the subsequent mouse received a decreased dose. Conversely, in the case of a negative response, the following mouse received an increased dose. Tests were conducted sequentially, with changes in mobility response transitioning between positive and negative until at least 7 crossover points were reached. Probit analysis was employed to determine the **ED50** (50% effective dose) and **ED95** (95% effective dose) of **C10**, along with their corresponding **CI** (confidence intervals). Sequence diagrams and dose-response curves were generated using GraphPad Prism 9 software.

Mobility recovery assessment in a mouse model: the trained mice for 4 days were *i.v.* administered with either 4 mg/kg or 6 mg/kg of **1b**, or saline, 30 seconds after administering 0.53 mg/kg **C10**. The mice were then placed on the rotor, and the time taken to move on the rotor for 30 seconds successfully was recorded.

### Pharmacokinetics studies

Male Kunming mice (30 ± 2 g) were used in the pharmacokinetic study. Drugs were injected through the tail vein of mice. Blood, liver, and kidney tissues were collected at certain intervals after administration with 6 mice at each time point. Blood samples were placed in heparin sodium tubes and centrifuged at 6000 rpm for 20 minutes at 4^ o^C to obtain plasma samples. Plasma, liver and kidney tissues were stored in a freezer at -80 ^o^C and frozen until analysis was performed. The drug concentrations were determined by Liquid chromatography-mass spectrometry (Waters ACQUITY UPLC/Xevo TQ-S). The Masslynx 4.1 software was used to obtain the peak area of the tested compounds and internal standards in each plasma sample and calculate the standard curve formula. The following pharmacokinetic parameters were calculated in each group using the DAS 3.2.8 software: C_max_ (maximal plasma level), t_max_ (time to peak concentration), AUC (0-48 h) (area under the curve between 0 and 48 h), AUC (0-∞) (area under the curve extrapolated until infinite), MRT (mean residence time), t1/2z (terminal elimination half-life), V_d_ (volume of distribution) and CL (total clearance). The detailed pharmacokinetics experiment procedure including materials and reagents, sample preparation, establishment and validation of analytical strategy, and LC-MS conditions are described in the Supporting Information.

### Statistical analysis

Statistical analysis was performed using PASW Statistics 18.0 and GraphPad Prism 9.5.1 software. One-way ANOVA was utilized for experiments involving more than two groups, while two-tailed, unpaired Student's t-tests were conducted for comparisons between two sample sets. Statistical significance was defined as P < 0.05. *P < 0.05, **P < 0.01, ***P < 0.001, ****P < 0.0001, ns indicates no statistically significant difference.

### Study approval

Female Balb/c mice aged 8-10 weeks were procured from Changsha Tianqin Biotechnology Co. Ltd and approved by Hengyang Medical School, University of South China. All mice had ad libitum access to food and water during the experiments. The animal experiments adhered to ethical standards, approved by the Institutional Animal Care and Use Committee of the Center (NO: USC2023XS107, USC2023XS130, USC2024XS037), following the guidelines of the Association for Assessment and Accreditation of Laboratory Animal Care International.

## Results and Discussion

### Binding behaviors between the amide naphthotubes with TC and C10

**Figure 2:**
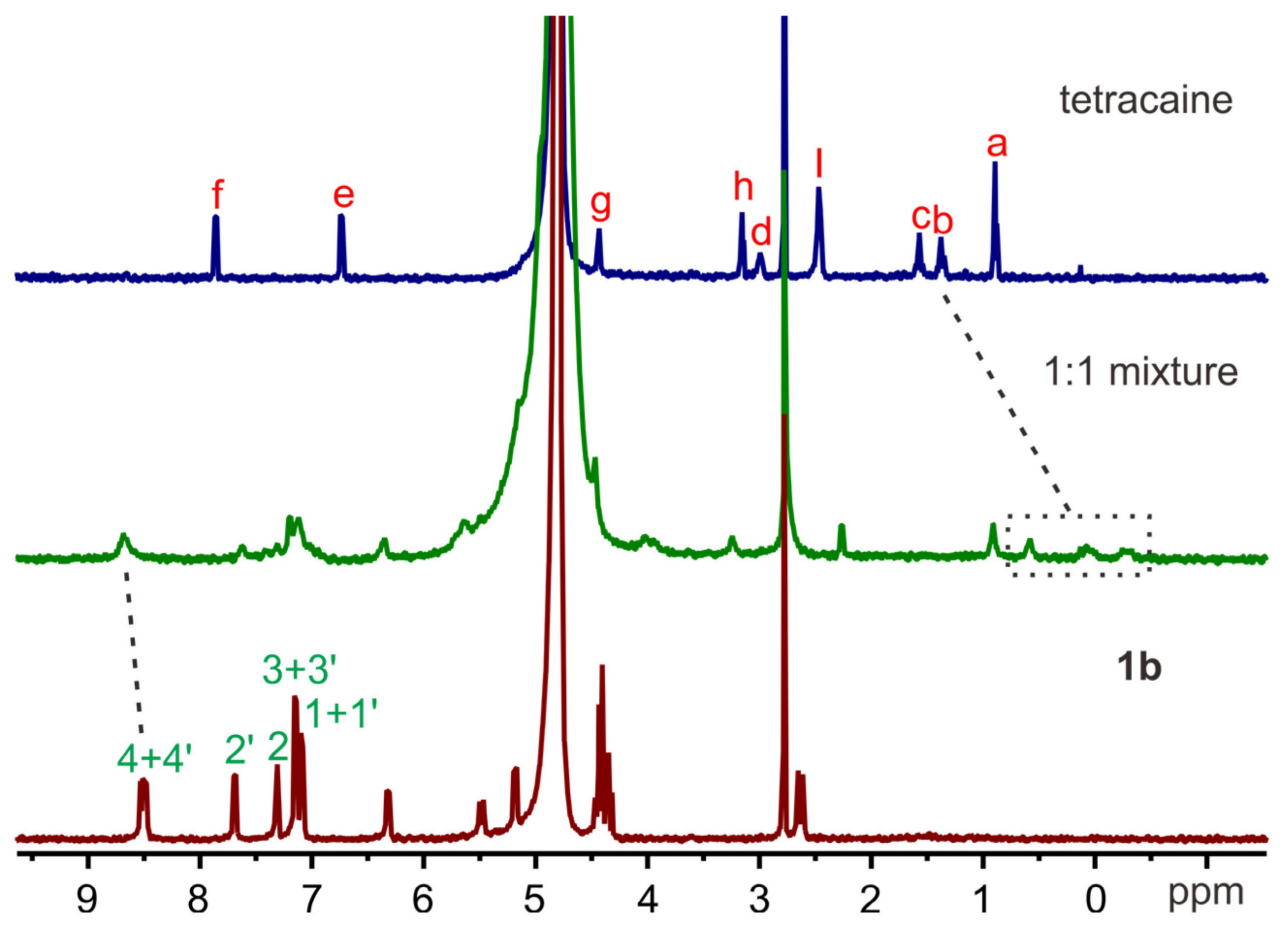
The ^1^H NMR spectra (500 MHz,0.5 mM, 298 K) of **TC** (top), **1b** (bottom) and their equimolar mixture (middle) in PB buffer (pD =7.4).

The complexation of amide naphthotubes with **TC** and **C10** in aqueous environments was initially investigated through ^1^H-NMR spectroscopy. All the complexes exhibit rapid exchange dynamics between the unbound and bound states at the NMR timescale, resulting in observed chemical shifts that represent an ensemble average of these states. Consequently, many signals become broad or disappear into the baseline. For the complex of the hosts with **TC** (Figures 2 and S1), the aromatic signals of the host experience broadening and obvious chemical shifts. Moreover, some guest signals are no longer detectable, while methylene signals of the guest molecules shift towards approximately 0 ppm. For the complex of the hosts with **C10** (Figures S2-S3), strongly shielded methyl signals between -1.5 ppm and -3.5 ppm confirmed the formation of the corresponding inclusion complexes. These NMR spectral alterations collectively provide evidence for the encapsulation of guest molecules within the host cavity.

**Figure 3:**
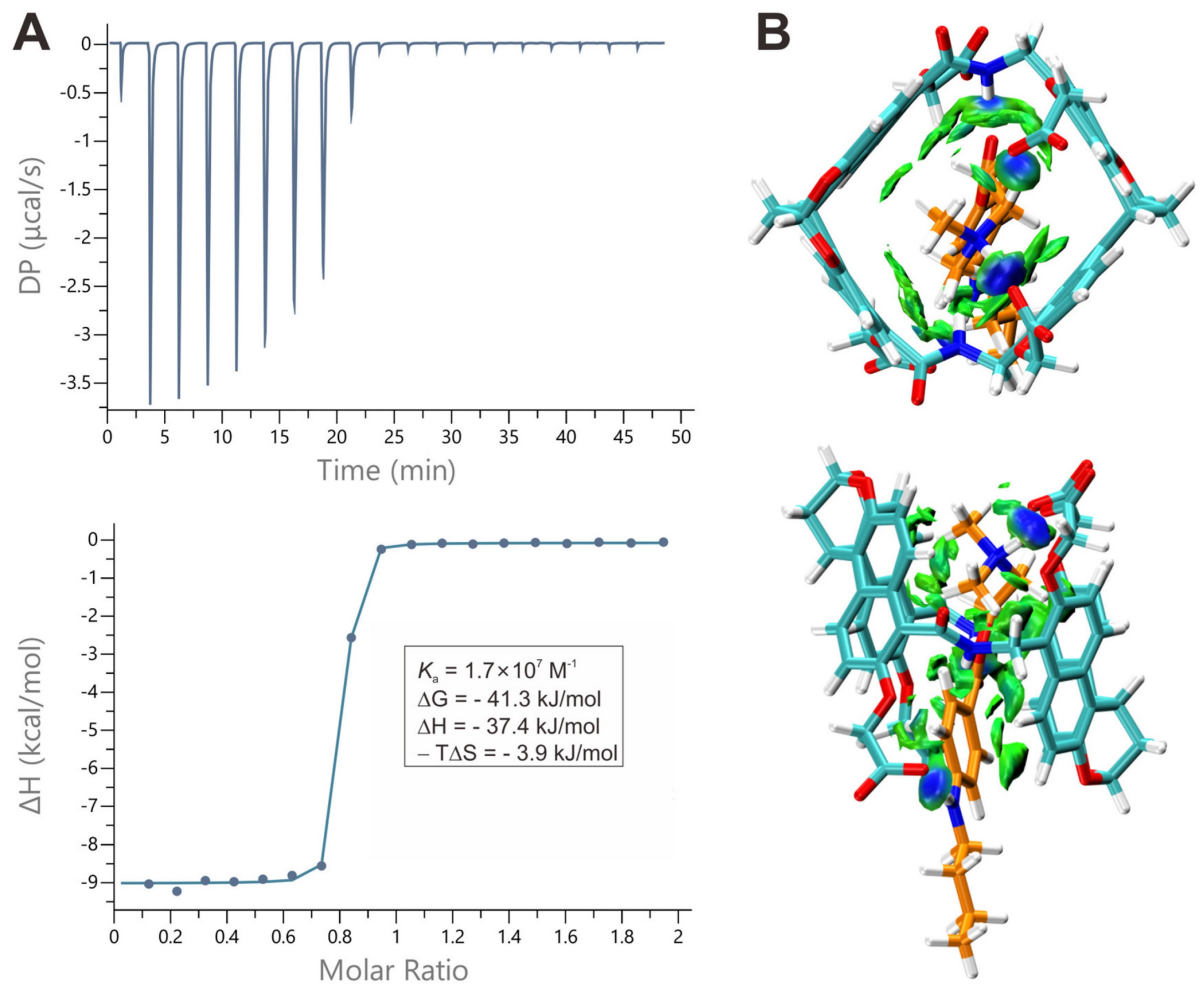
** Binding behaviors of amide naphthotube and guests**. (**A**) top: Raw ITC data obtained from titration of **1b** with **TC** in water at 25 ^o^C, bottom: apparent reaction heat obtained from the integration of calorimetric traces and fitted using the “one set of binding sites” model. (**B**) Energy-minimized structures and independent gradient models of** TC**@**1b** obtained by DFT (ωB97xd/(ma)-def2-SVP) calculations.

After confirming the inclusion binding of guests within hosts **1a** and **1b** by ^1^H NMR spectroscopy, we thus conducted ITC measurements to determine the binding thermodynamics for all complexes (Figures 3A, S4-S7). By titrating the solutions of the hosts with the guests and using the "one set of binding sites" model, we extracted the values of *K*a, ΔH, -TΔS and the binding stoichiometry, presented in Table 1. The binding constants are all higher than 10^6^ M^-1^ and the binding events are predominantly driven by favorable changes in enthalpy, which are attributed to the non-classical hydrophobic effects 57. This effect involves the release of cavity waters with incomplete hydrogen bonding into the surrounding solvent upon complexation 58. These association constants are much higher when compared to other classic molecular hosts, whose affinities are lower than 10^5^ M^-1^ and 10^7^ M^-1^ toward **TC** and **C10** 59, 60, 61, respectively.

To investigate the binding strength of amide naphthotube with **TC** and **C10** under conditions mimicking biological environments, additional ITC experiments of **1b** were conducted at 37 ^o^C and in FBS, as **1b** is a stronger binder than **1a** (Figures S8-S11). At 37°C, the binding constants of **1b** with **TC** and **C10** show a slight decrease compared to 25°C, yet remains approximately 10^7^ M^-1^. Moreover, the changes of ΔG for complex formation exhibit minimal variation (<1.5 kJ mol^-1^) across temperatures, contrasting with more pronounced alterations in ΔH and -TΔS (ca. 2.2-4.4 kJ mol^-1^). Notably, the opposing changes in ΔH and -TΔS compensate each other, thereby enabling the maintenance of strong binding affinities even at elevated temperatures, demonstrating an enthalpy-entropy compensation effect. Serum contains high concentrations of salts and diverse small biomolecules 62. For example, some quaternary ammonium salts such as acetylcholine and choline present in the blood may bind the hosts competitively. The binding affinities between **1b** and acetylcholine and choline were determined by NMR titrations (Figures S12-S15), and the values are four orders of magnitude lower than those for** TC** and **C10**. The binding constants of **1b** towards **TC** and **C10** in 100% FBS (*K*_a_ = 1.74 × 10^5^ M^-1^, 1.44 × 10^5^ M^-1^) are two orders of magnitude lower than that in water. This reduction is considered moderate, given the presence of a myriad of intricate components within the serum. Therefore, the strong binding of **1b** under mimicking physiological conditions provides a foundational assurance for its detoxification efficacy in vivo.

DFT calculations for all the complexes at the level of theory of ωB97XD/def2-SVP (ma-def2-SVP basis set was used for anionic parts) in water (PCM) were then performed to elucidate the detailed interactions responsible for the strong binding (Figures 3B, S16-S18). Generally, both **TC** and **C10** are well encapsulated inside the cavity of **1a** and **1b**. Independent gradient model based on Hirshfeld partition (IGMH) analysis reveals that C-H⋅⋅⋅π, N-H⋅⋅⋅π, π⋅⋅⋅π and electrostatic interactions between the guests and the hosts are involved in the complexes. Moreover, the optimized structures provide insights into the differences in binding constants of **1a** and **1b**. For **TC**, the deeper cavity of **1b** results in a more enclosed inclusion than **1a**, thus a slightly higher binding constant for **1b** than **1a** is observed. Moreover, there are two representative conformers for the host-guest complex for **1a** and **TC**, the DFT calculations show the conformer with more favored electrostatic interactions of carboxylate groups with the positive Protonated tertiary amine is more energetically stable (Figure S16). Generally, **1a** and **1b** are very potent for binding** TC** and **C10** but **1a** is a slightly weaker host than **1b**, so the latter biocompatibility and antidotal tests are generally based on **1b**.

### Biocompatibility evaluation of 1b in a mouse model

**Figure 4:**
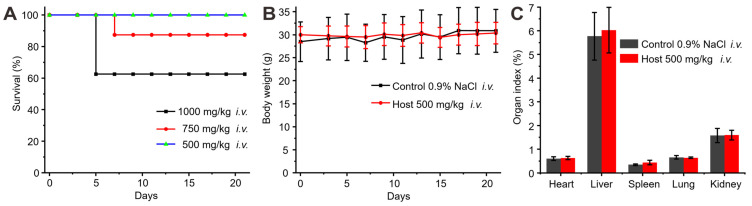
** Toxicity assessment of *i.v.* administration of 1b**. (**A**) Survival rates of mice after *i.v.* administration of varying doses of **1b**. (**B**) Weight changes in mice after* i.v.* administration of **1b** (500 mg/kg). (**C**) Organ indices of the mice 21 days after *i.v.* administration of **1b**.

The biocompatibility of **1b** was previously assessed in vitro and showed minimal cytotoxicity at concentrations up to 0.3 mM 49. However, a systematic examination of its in vivo toxicity has been lacking. In this study, the in vivo biocompatibility of **1b** was evaluated in mice following *i.v.* administration by monitoring changes in body weight, examining organ indices, hematology parameters, hepatic function markers, and conducting histopathological analysis of major organ tissues. The assessment of a drug's hemolytic effects is critical for ensuring its safety for *i.v.* administration. To evaluate the hemolytic activity of **1b**, a Red Blood Cell Hemolysis test was conducted using blood samples obtained from Sprague-Dawley. The results indicated that the RBC hemolysis ratio for **1b** remained below 0.5% across a concentration range of 10 to 800 µM (Figure S19), thus suggesting minimal hemolytic activity on erythrocytes. After confirming the minimal hemolytic activity of **1b**, we began our next step, which involved assessing the in vivo biocompatibility of **1b**. We initially determined the maximum tolerable dose of **1b** in mice through *i.v.* administration (Figure 4A). Administering **1b** at doses of 1000 mg/kg or 750 mg/kg resulted in approximately 40% or 10% mortality 4-8 days post-administration. After reducing the dose to 500 mg/kg, all the mice remained alive and showed no unusual behaviors or signs of illness throughout the 21-day experiment. Subsequent examination revealed a marginal reduction in body weights during the initial 7 days post-administration, followed by a consistent rise thereafter, as elucidated in Figure 4B. Notably, evaluation of organ indices in mice sacrificed on Day 21 following *i.v.* injection of **1b** demonstrated no discernible disparities as compared to the control group, as illustrated in Figure 4C.

**Figure 5:**
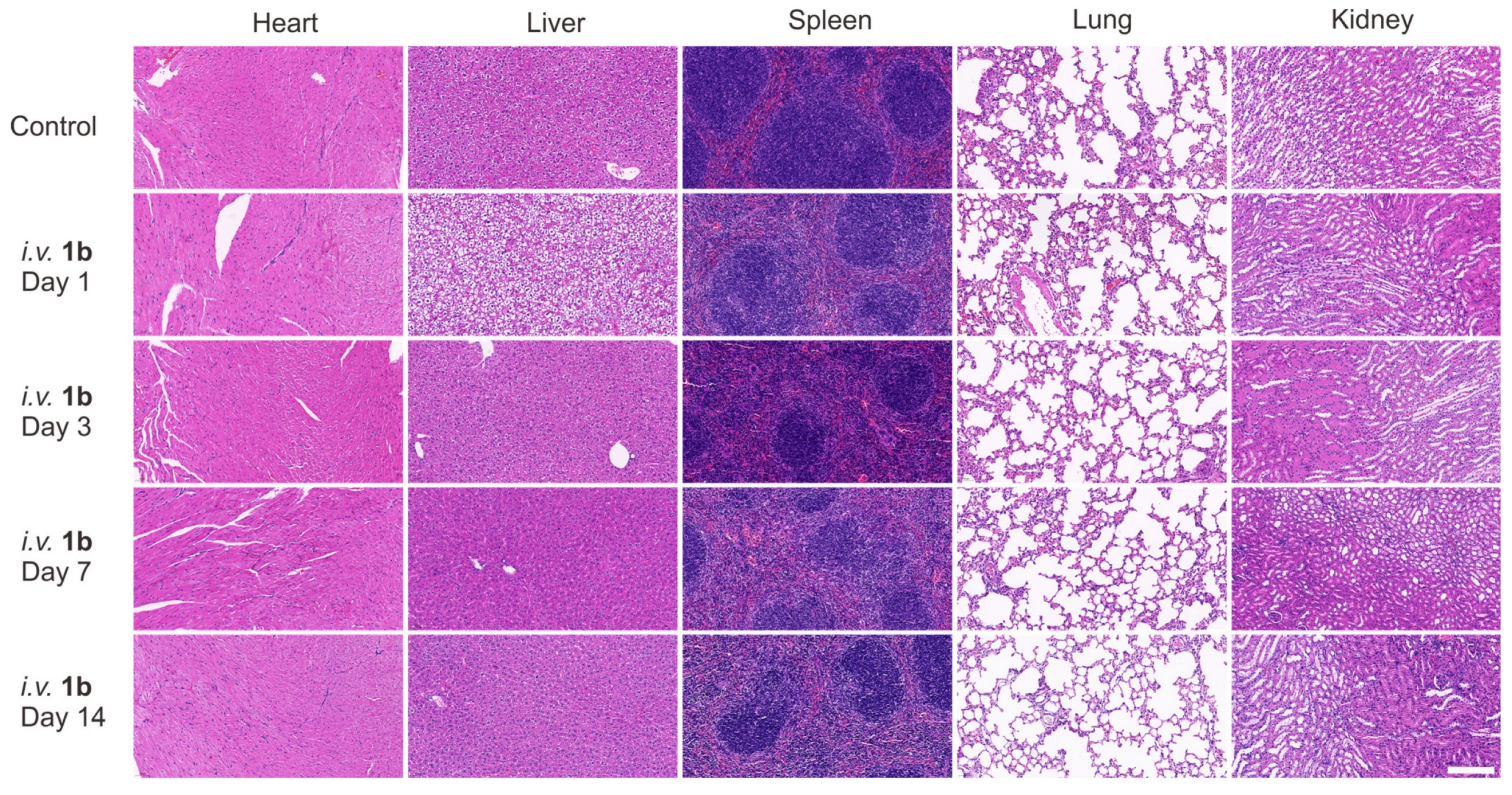
** H&E stained sections of major organs with intravenous administration of 1b (100 mg/kg).** Mice were sacrificed on day 1, 3, 7, 14 after the *i.v.* administration of **1b**. Scale bar = 200 μm.

**Figure 6:**
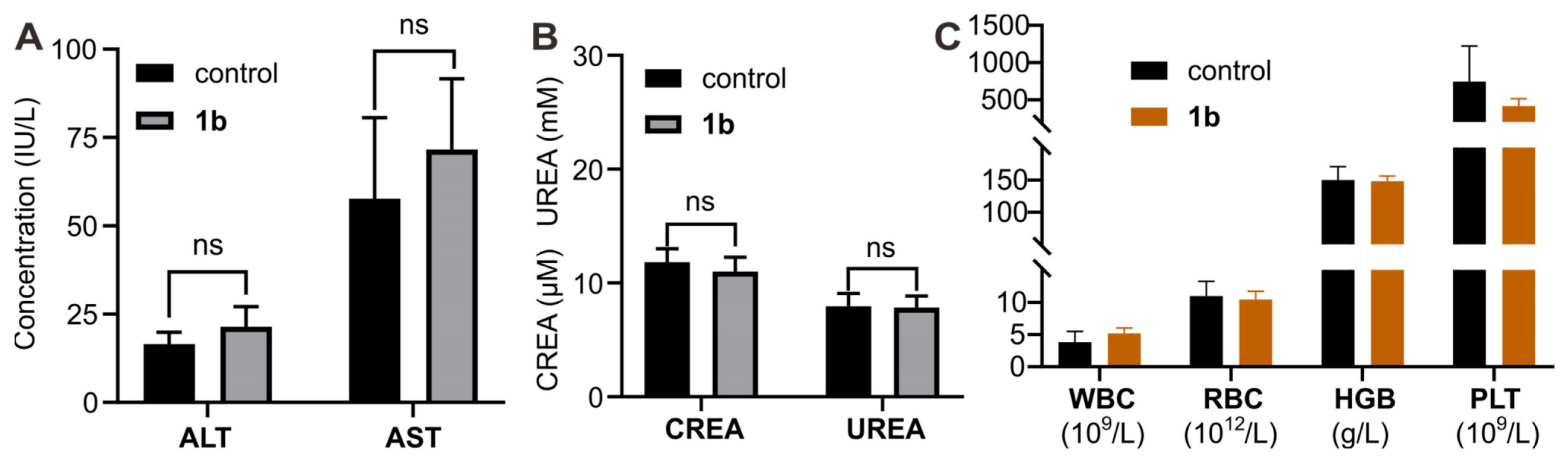
** Toxicity evaluation of 1b with *i.v.* administration**. **(A)** Hepatic and renal (**B**) function markers test on the blood samples collected from the mice on Day 14 after *i.v.* administration of **1b**. (**C**) Hematological parameters of blood from mice that were sacrificed on Day 14 after *i.v.* administration of **1b**.

Furthermore, histological analysis was conducted following *i.v.* administration of **1b** at 100 mg/kg doses (maximum possible dosage estimated according to the literature). Hematoxylin and eosin H&E staining was employed to assess tissue samples obtained through the mice sacrificed at 1, 3, 7, and 14 days post-administration (Figure 5). Generally, the histopathological sections of the majority of the major organs in the mice did not display discernible signs of injury or inflammatory cell infiltration, consistent with the findings in the control group. Specifically, mild hepatocyte edema was observed on the first day following **1b** administration, indicative of a certain degree of associated hepatotoxicity. Nonetheless, the subsequent reduction in hepatic edema by the third day suggests the liver damage caused by **1b** was reversible. Furthermore, the hepatic and renal function tests conducted on day 1 (Figure S20) and day 14 (Figures 6A and 6B) after **1b** injection revealed no statistically significant differences in aspartate aminotransferase (AST), alanine aminotransferase (ALT), creatinine (CREA), and urea nitrogen (UREA) levels compared to the control group, indicating minimal impact on the hepatic and renal functions. Subsequent analysis of typical hematological parameters in the blood revealed values within the normal range, comparable to those of the control group, providing further evidence of the low cytotoxicity of **1b** (Figure 6B). Taken together, these findings indicate the high *i.v.* biocompatibility of **1b** in mice at a dose as high as 100 mg/kg, as demonstrated by the absence of toxicity in our systemic evaluations.

**Figure 7:**
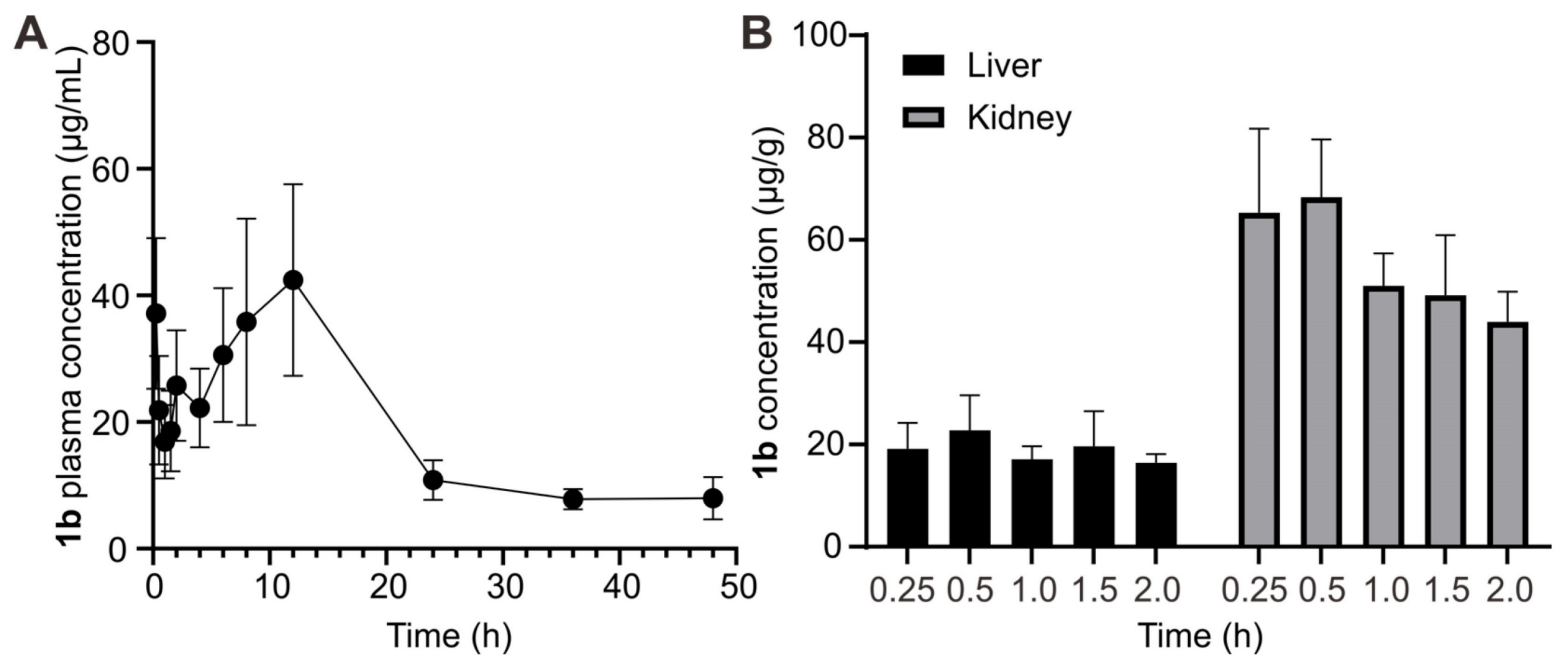
** Pharmacokinetics of 1b. (A)** Plasma concentration-time plots of **1b** after *i.v.* administration. **(B) 1b** concentrations in the liver and kidney at different time points.

In addition to toxicity assessments evaluating the safety of **1b**, understanding its plasma pharmacokinetic profile in *vivo* is crucial to assessing its clinical potential as a scavenger of **TC** and **C10**. Following *i.v.* administration (80 mg kg^-1^), plasma concentration of **1b** at various intervals was measured based on UPLC-Xevo TQ-S MS/MS. The concentration-time profile was depicted in Figure 7A, and pharmacokinetic parameters were determined using non-compartmental modeling analysis with DAS 3.2.8 software and summarized in Table 2. After *i.v.* administration, the maximum plasma concentration (C_max_) was 43.02 μg/L. The area under the plasma concentration-time curve (AUC_0-∞_) value was 999.15μg/L/h. The half-life (t_1/2_) of **1b** was 14.52±6.32 h. The relatively long t_1/2_ of **1b** ensures that drugs captured by **1b** are less prone to re-occurrence of toxicity or effects. The established LC-MS protocol also analyzed the tissue distribution of 1b in the liver and kidney. The result (Figure 7B) shows that the concentration of **1b** in the kidney is higher than in the liver at all times. Meanwhile, the highest content was reached at 0.25 h in those two organs, hinting that **1b** could be easily transferred into tissues.

### In vitro relieving effects of 1b on TC-poisoning

**Figure 8:**
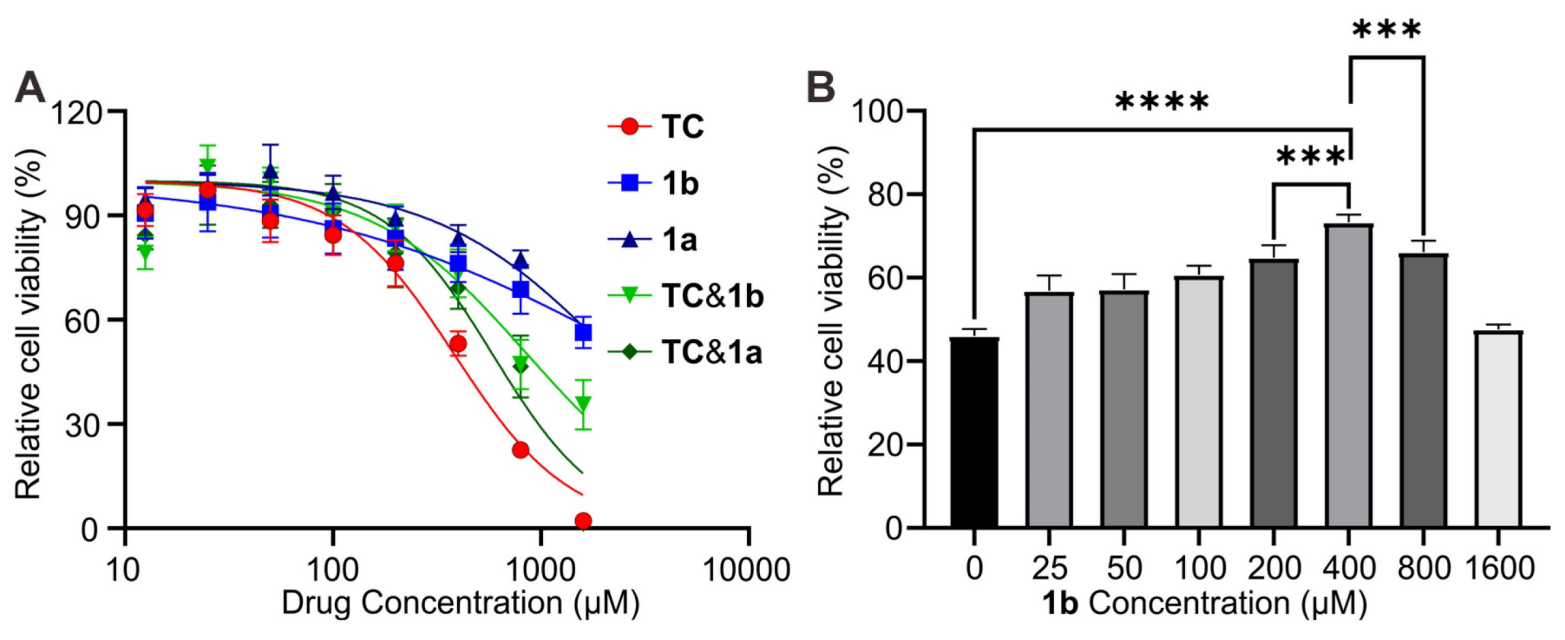
** In vitro relieving effects of 1b. (**A) Cytotoxicity of **TC**, **1a**, **1b**, **TC**@**1a**, **TC**@**1b** against AC-16 cells after incubation for 24 h. (B). In vitro cytotoxic effect on AC16 cells after incubation for 24 h with fixed **TC** (400 μM) and different proportions of **1b** at different concentrations. Data were represented as mean ± SD from six independent experiments.

The capacity of **1b** as a specific antidote for reversing **TC** toxicity was first investigated in vitro in Human myocytes AC16 cell, as **TC** is known to induce cardiac toxicity. The cytotoxicity of the drug was evaluated using the CCK-8 assay method. Figure 8A shows the cell viability after incubation of AC16 for 24 hours with these compounds at concentrations up to 1600 μM. The IC_50_ values were determined to be 383.7 μM, 2189 μM, and 2961 μM for **TC**, **1a**, and **1b**, respectively. Furthermore, when **TC** was mixed with **1a** or **1b** in a 1:1 ratio, the toxicity was effectively reduced, resulting in higher IC_50_ values of 586 μM and 862 μM for **TC**@**1a** and **TC**@**1b**, respectively. These findings provide evidence for the potential detoxification effect of the hosts on **TC**. We further investigated the impact of different dosages of **1b** on reducing cellular toxicity with **TC** concentration fixed at 400 μM. The results demonstrated that the cell survival rate was the highest at a 1:1 ratio, indicating the optimal detoxification effect (Figure 8B).

### Improved survival in a lethal TC-poisoning mouse model

**Figure 9:**
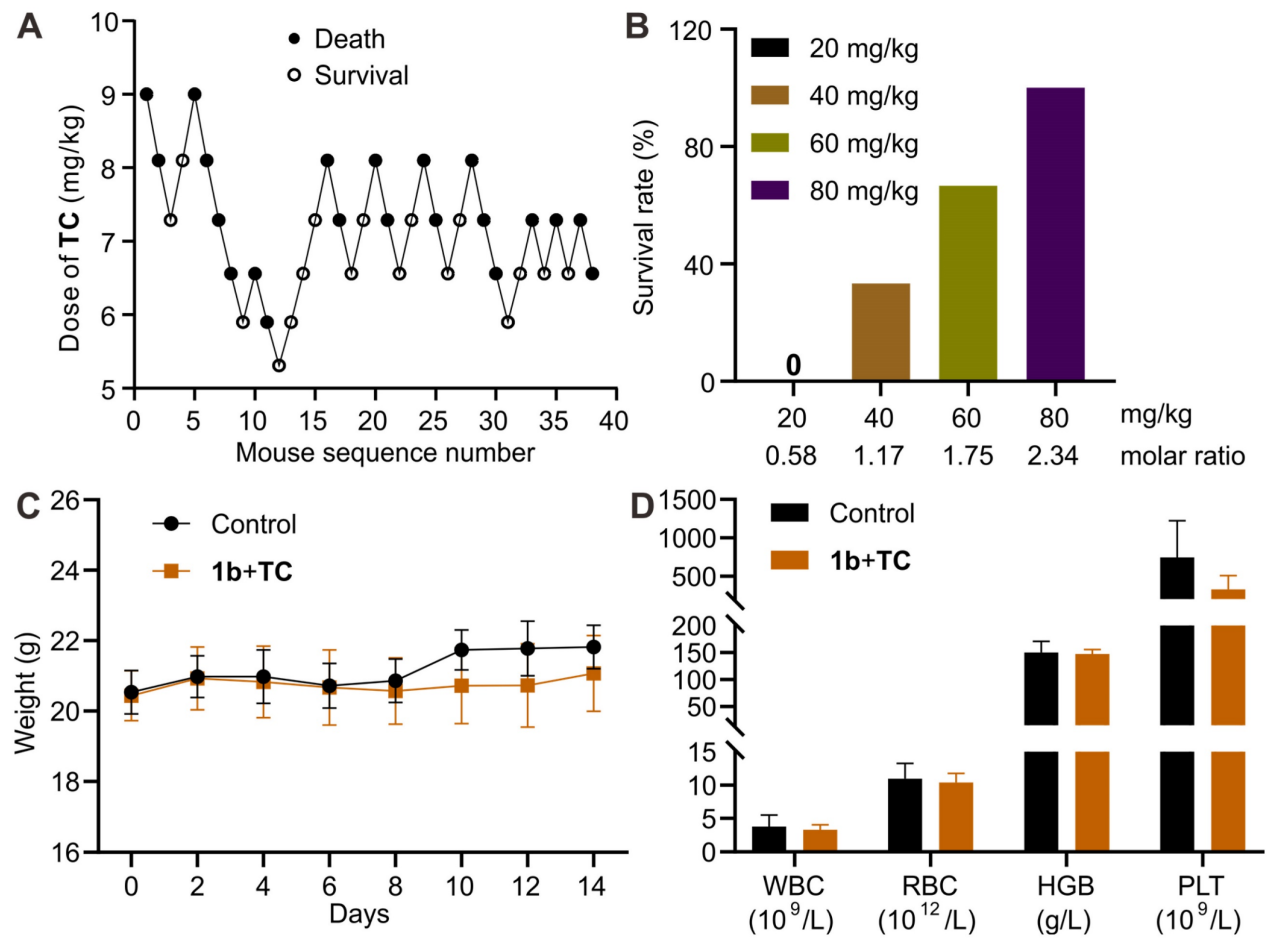
** 1b reversed TC-induced death in a mouse model**. **(A)** Consecutive **TC** concentration following the Dixon up-and-down method for determining lethal dose. **(B)** Mice survival rates when treated with varying doses of **1b** immediately after receiving a lethal dose of **TC**. **(C)** Changes in body weight following treatment with **1b** after *i.v* administration of 9 mg/kg **TC**. **(D)** Hematological parameters of mice 14 days after treatment with **1b** following the *i.v.* administration of a lethal dose of **TC**.

After confirming the detoxification effect of **1b** in vitro, we performed experiments to assess its ability to reverse the **TC** toxicity in vivo. To select an appropriate dose of **TC** for this study, we first estimated its **LD_50_** and **LD_95_** using Dixon's up-and-down method (Figure 9A). The initial animal received a dose of 9.0 mg/kg. Subsequent dosages for the following animals were adjusted based on whether the preceding animal survived. Higher doses were administered if survival occurred and lower doses if the animal perished. Mortality rates were recorded for each dosage group. Probit analysis (Figure S21) was used to calculate the **LD_50_** and **LD_95_** values of **TC** as 6.9 mg/kg (95% **CI**, 6.1-7.5 mg/kg) and 8.7 mg/kg (95% **CI**, 7.9-12.9 mg/kg), respectively. Based on these findings, we chose the lethal dose of 9.0 mg/kg to evaluate the antidotal effects of **1b** on **TC**-induced mortality. Mice received varying doses of **1b** immediately after the *i.v.* administration of **TC**. As depicted in Figure **9B**, the injection of **1b** notably decreased **TC**-induced mortality, with all mice surviving at a **1b** dose of 80 mg/kg (**1b**/**TC** = 2.34). This suggests that **1b** can completely reverse **TC**-induced death when administered at a dose higher than 2.34 equiv. of **TC**. We then assessed the mice that survived after treatment with **1b** following a lethal dose of **TC**. Their body weights remained similar to the control group during the 14-day follow-up period (Figure **9C**), and their blood parameters indicated that values were comparable between the treatment and control groups (Figure **9D**).

### Reversal of TC-induced alterations in plasma membrane potential and restoration of blocked sodium channels in vitro

**Figure 10:**
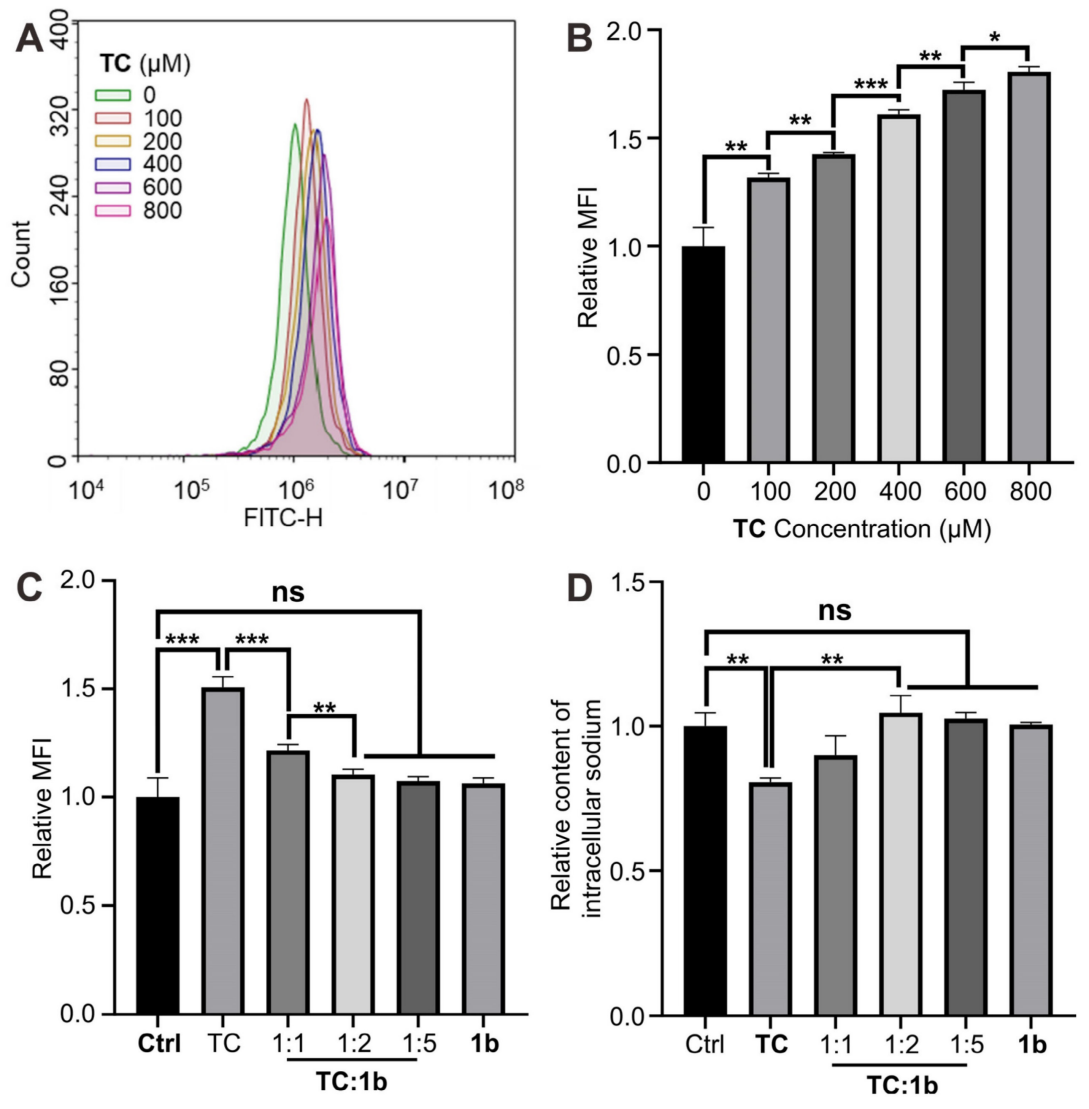
** 1b reversed changes in plasma membrane potential and reduced intracellular sodium loss in vitro.** (**A** and **B**) Typical flow cytometry curves (A) and quantitative analysis (B) for determining the plasma membrane potential of AC16 cells treated with **TC** for 10 minutes. (C) Quantitative analysis of the plasma membrane potential of AC16 cells treated with TC, **1b** or co-incubated with **TC** and **1b** at various molar ratios for 10 min. (D) Relative intracellular sodium content of AC16 cells treated with TC, 1b, or co-treated with **TC** and 1b at various molar ratios for 10 minutes. Data were represented as mean ± SD from three independent experiments.

Voltage-gated Na^+^ channels are dynamic transmembrane proteins essential for initiating action potential upstrokes in excitable membranes. Local anesthetics selectively bind to specific sites within these channels, blocking Na^+^ currents and preventing normal depolarization 63. This alters the plasma membrane potential, leading to conduction block and reducing excitability in neurons, cardiac tissue, and the central nervous system. To explore reversal mechanisms at the cellular level, we employed the Human myocytes AC16 cell line, on which Na^+^ channels are very easily blocked by local anesthesia. Using a potential-sensitive fluorescent probe, we measured relative plasma membrane potential changes, observing increased cellular fluorescence after the sodium channel was blocked (Figures 10A-10B). The results demonstrate a significant rise in mean fluorescence intensity during **TC** exposure. Co-incubation with **TC** and **1b** at molar ratios of 1:1 and 1:2 respectively markedly reduced mean fluorescence intensity (MFI) compared to cells treated with **TC** alone, indicating that **1b** reverses **TC**-induced plasma membrane potential changes at the cellular level and restored the normal depolarization of cells (Figure 10C). Further investigations revealed that **1b** effectively restores **TC**'s blocking of sodium ion channels (Figure 10D). Exposure to **TC** (400 µM) for 10 minutes resulted in approximately 20% intracellular sodium loss, which was fully reversed by co-incubation with **TC** and **1b** (1:2). These results highlight that **1b** restores Na^+^ channels blocked by **TC**. The cells return to normal action potential and excitation conduction.

### Improved survival in a lethal C10-poisoning mouse model

**Figure 11:**
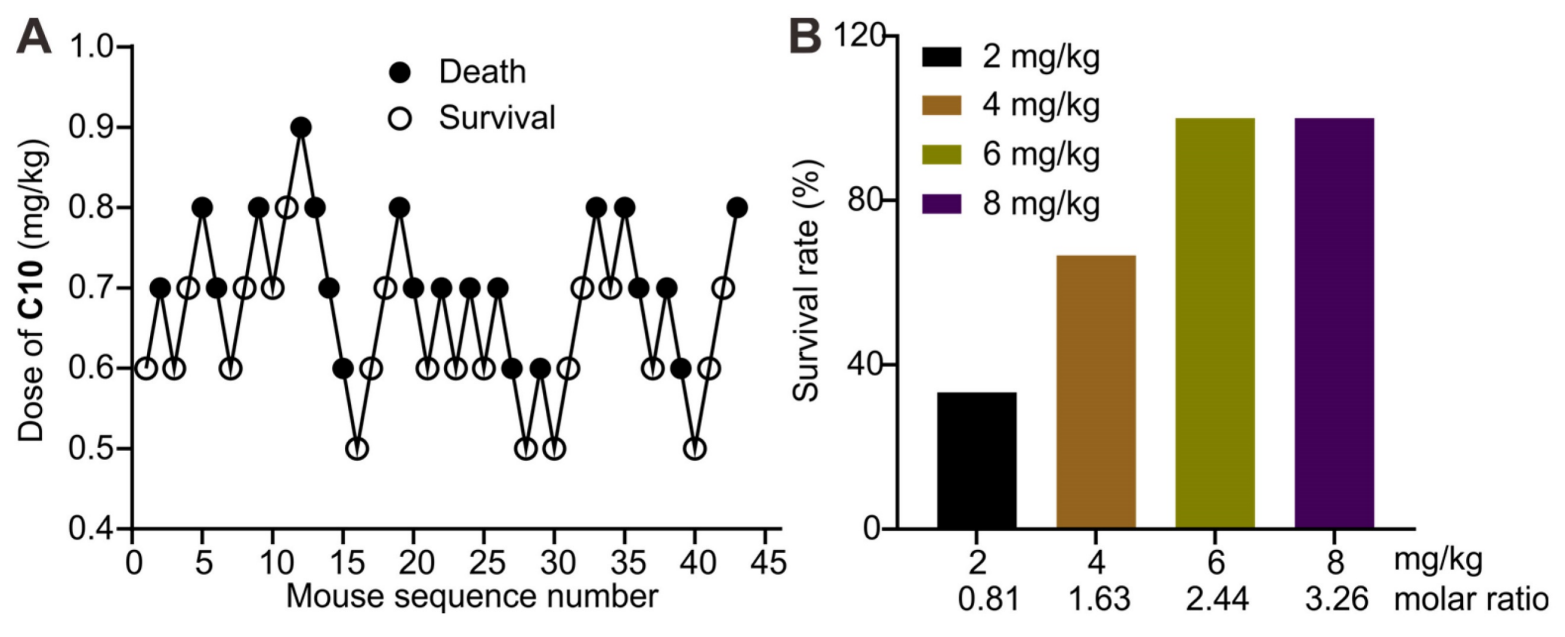
** 1b reversed C10-induced mortality in mice**. **(A)** Consecutive **C10** concentration following the Dixon up-and-down method for determining lethal dose. **(B)** Survival rates of mice treated with various doses of **1b** immediately after the *i.v.* administration of a lethal dose of **C10** (0.9 mg/kg).

The application of **1b** as a supramolecular sequestration agent for then conducted neuromuscular blocking agent **C10**. The evaluation of **LD_50_** and **LD_95_** of **C10** in mice was performed to determine an appropriate dose for this preclinical study. Dose-mortality data for each mouse, obtained by the up-and-down method, are shown in Figure 11A. Probit analysis (Figure S22) yielded an **LD_50_** of 0.69 mg/kg (95% **CI**, 0.65-0.75 mg/kg) and **LD_95_** of 0.86 mg/kg (95% **CI**, 0.78-1.11). The lethal dose of 0.9 mg/kg was then used to evaluate the antidotal effects of synthetic receptors. Administering **1b** at different doses immediately after the **C10** injection revealed its potential to fully reverse **C10**-induced mortality at doses higher than 2.44 equiv. of **C10** (Figure 11B). This effect is likely due to **1b**'s specific binding and sequestration of **C10** in the blood and neuromuscular junctions, leading to the dissociation of **C10** from the nicotinoyl acetylcholine receptor.

### Reversal of paralysis in a mouse model

**Figure 12:**
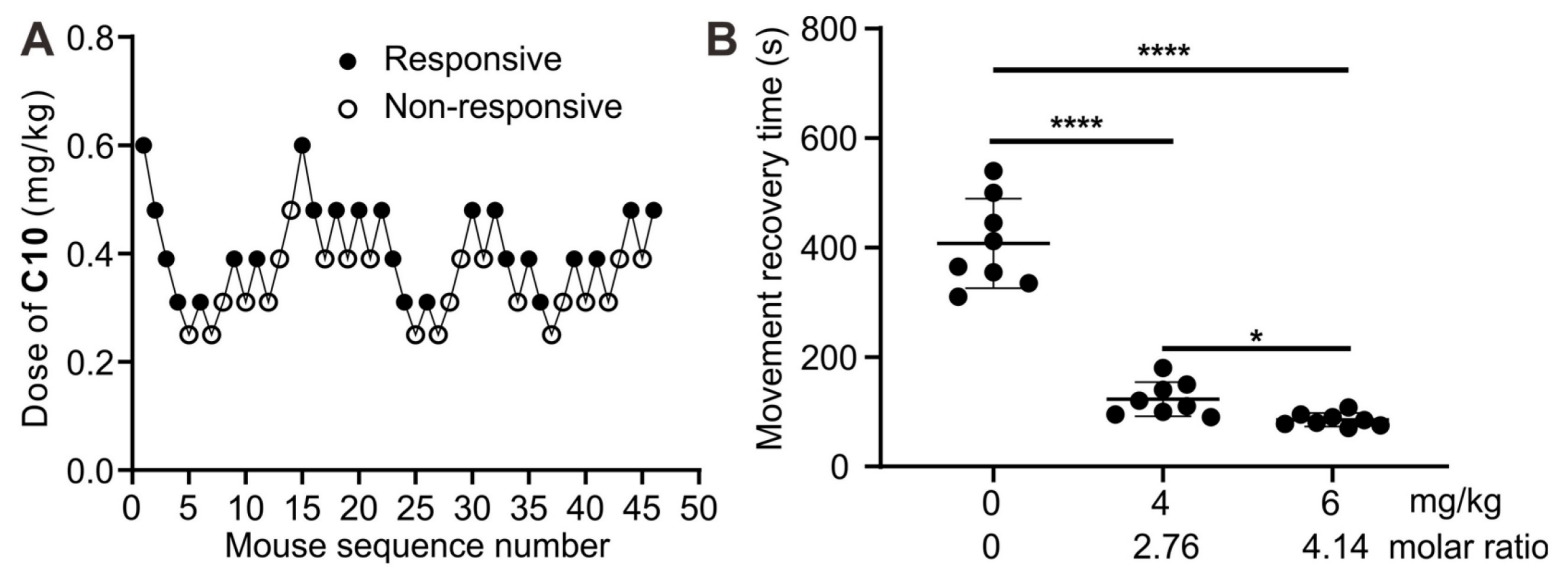
** Reversal of paralysis in a mouse model. (A)** Consecutive **C10** concentration following the Dixon up-and-down method for determining effective dose. **(B)** movement recovery time of the mice when *i.v.* administered with **1b** (4 mg/kg or 6 mg/kg) at 30 s after the administration of **C10** (0.53 mg/kg).

Subsequently, an assessment was conducted to investigate the potential of **1b** to reverse the paralysis caused by **C10** and expedite mobility restoration in a murine model. The half-effective dose (**ED50**) and 95% effective dose (**ED95**) of **C10** for inducing muscle paralysis were determined using the up-and-down method (Figure 12A). According to probit analysis (Figure S23), the ED50 of C10 was 0.37 mg/kg (95% **CI**, 0.32-0.42 mg/kg), and the ED95 was 0.53 mg/kg (95% **CI**, 0.46-0.76 mg/kg). Therefore, we selected 0.53 mg/kg to assess the reversal effects of **1b** on **C10**-induced paralysis. Immediately after *i.v.* administration of **C10** to the mice, paralysis ensued, preventing movement on an accelerating rotor, necessitating prompt removal. Due to the in vivo metabolism of **C10**, the mice gradually regained mobility within minutes, allowing continuous movement on the rotor for over 30 seconds, similar to control mice. The average recovery time was approximately 402.8 seconds for mice given **C10** (Figure 12b). In contrast, the average recovery time was reduced to 123.1 seconds and 85.1 seconds when mice were given **1b** at doses of 4 mg/kg and 6 mg/kg, respectively, 30 seconds after the administration of **C10**. This observation was attributed to the complexation of **C10** by **1b** in the blood, thereby expediting neuromuscular recovery. These findings demonstrate the potential of **1b** in reversing protracted paralysis induced by **C10**, especially in instances of misuse or overdose in clinical settings.

### Pharmacokinetic experiments

**Figure 13:**
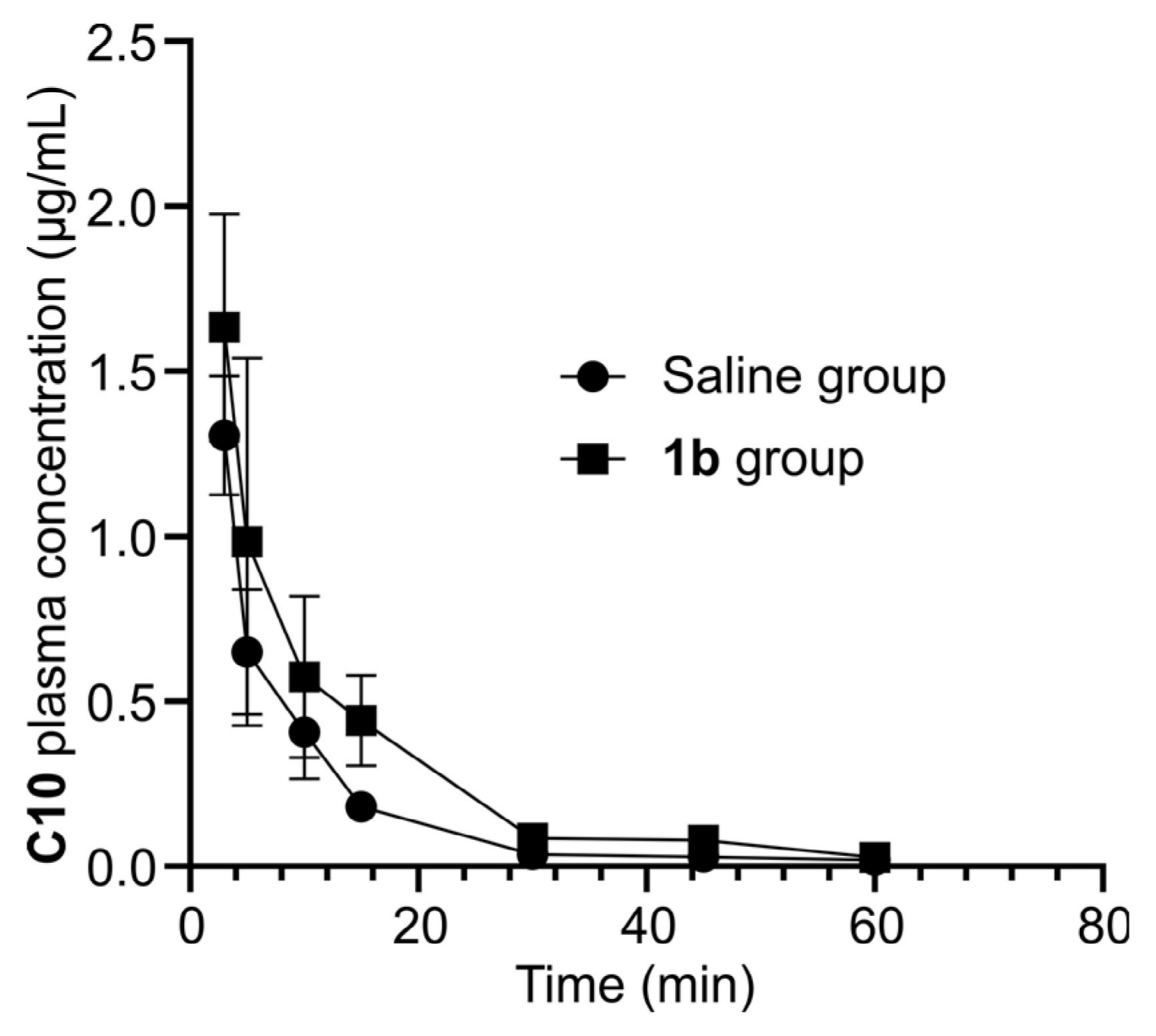
** Plasma pharmacokinetics of C10. C10** plasma concentration against time for both the saline- and **1b**-treated groups.

Furthermore, we conducted a quantitative analysis of **C10** concentration in blood to elucidate the potential mechanism of action of reversal agent **1b**. Sixty seconds after intravenous injection of **C10**, KM mice were randomly assigned to receive either **saline** or **1b**. Blood samples were collected at predetermined intervals to determine the concentration of **C10** using LC-MS. The plasma concentration-time profiles of **C10** are depicted in Figure 13, and the key pharmacokinetic parameters are summarized in Table 3. The non-compartmental model was employed to characterize the pharmacokinetics of both formulations. **C10** levels exhibited rapid decline during the initial 0.2 hours post-dose for both groups. After treatment with **1b**, the maximum plasma concentration (C_max_) and the area under the plasma concentration-time curve (AUC_0-∞_) increased from 1305.40 ± 179.41 to 1633.02 ± 344.88 ng/mL, and from 293.66 ± 27.97 to 414.10 ± 80.89 ng/mL/h, respectively. Whereas, the total body clearance for the saline-treated Group and **1b**-treated Group significantly decreased from 1.54 ± 0.16 to 1.12 ± 0.22 mL/h/kg. The pharmacokinetic findings generally indicate an increase in total **C10** concentrations following administration of **1b**. These observations are consistent with the pharmacokinetic model proposed for reversing neuromuscular blockade by Sugammadex 64, 65, 66. Thus, the reversal of **C10** by **1b** follows a pharmacokinetic mechanism: **1b** binds specifically and with high affinity to **C10** through encapsulation in the plasma. Free **C10** molecules in the plasma are sequestered by **1b**, resulting in a rapid decrease in the concentration of free **C10** in the plasma. This establishes a concentration gradient between free **C10** in the neuromuscular junction and the plasma, causing free **C10** molecules to migrate back to the plasma where they are encapsulated by **1b**. Consequently, this process leads to a swift reversal of the **C10** blockade.

## Conclusions

In summary, we have investigated the molecular recognition properties of amide naphthotubes toward **TC** and **C10** using a combination of ITC and DFT calculations. **1b** possessed strong binding ability towards **TC** and **C10** with *K*_a_ values of 1.89×10^7^ M^-1^ and 1.01×10^7^ M^-1^. The evaluation of the safety and biocompatibility of **1b** in mice via *i.v.* administration suggests a good biocompatibility profile of **1b** in vivo. Intravenous injection of **1b** could contribute to reverse overdose **TC** or **C10**-induced mortality and reverse the NMB effect of **C10** in mice models via direct host-guest encapsulation through a pharmacokinetics approach. Our systemic studies suggested that **1b** may be used as an in vivo sequestrant for toxicant poisoning, offering a prospective clinical approach for mitigating the adverse effects of local anesthetics.

More generally, macrocycles with endo-functionalized cavities have several important implications: (a) This study marks the first comprehensive evaluation of the safety and biocompatibility of amide naphthotube when administered intravenously in mice. The favorable safety profile observed suggests that amide naphthotube holds promise for further research and potential clinical applications in the field of biomedicine. (b) Local anesthetics are commonly utilized for managing postoperative pain and treating various chronic pain conditions. However, the risk of local anesthetic systemic toxicity poses a potentially life-threatening concern as their use in healthcare settings grows. Our systemic studies indicate that supramolecular hosts have the potential to serve as supramolecular antidotes for mitigating the adverse effects of local anesthetic drugs, thus offering a promising clinical approach for the treatment of local anesthetic systemic toxicity. (c) Although decamethonium is no longer utilized in clinical practice, it was previously extensively employed and might be reintroduced with the availability of appropriate antagonists. (d) The development of host systems as sequestrants for more complex guests beyond hydrophobic cations and to withstand the effects of physiological salt and serum proteins remains a challenge in the field of supramolecular sequestrants 18. The distinctive feature of amide naphthotube, which combines hydrogen-bonding sites within a hydrophobic cavity, suggests exceptionally strong binding not only with highly hydrophobic guest molecules but also potentially with hydrophilic molecules, thereby opening up new possibilities for supramolecular sequestration agents. In conclusion, we believe that this unique macrocyclic receptor enhances the repertoire of supramolecular sequestrants and holds promise for diverse future applications.

## Supplementary Material

Supplementary figures, materials and methods, and tables.

## Figures and Tables

**Table 1:** Association Constants and Thermodynamic Parameters as Determined by ITC Titrations.^a^

Guest	Host	Solvent	Temperature	*K*_a_ (M^-1^)	ΔG (kJ/mol)	ΔH (kJ/mol)	-TΔS (kJ/mol)
**TC**	**1a**	water	25 ^o^C	(1.32 ± 0.10) × 10^7^	-40.7	-32.9	-7.8
	**1b**	water	25^ o^C	(1.89 ± 0.24) × 10^7^	-41.5	-37.1	-4.4
	**1b**	water	37^ o^C	(1.40 ± 0.04) × 10^7^	-42.4	-40.2	-2.2
	**1b**	FBS	25 ^o^C	(1.74 ± 0.17) × 10^5^	-29.9	-23.1	-6.8
**C10**	**1a**	water	25 ^o^C	(5.73 ± 0.16) × 10^6^	-38.6	-21.0	-17.6
	**1b**	water	25 ^o^C	(1.01± 0.02) × 10^7^	-40.0	-23.9	-16.1
	**1b**	water	37^ o^C	(9.56 ± 1.33) × 10^6^	-41.4	-28.1	-13.3
	**1b**	FBS	25^ o^C	(1.44 ± 0.11) × 10^5^	-29.5	-15.3	-14.2

^a^ All the titration experiments were repeated twice, and the averaged values with standard deviations are reported here.

**Table 2:** Main pharmacokinetic parameters of **1b** after *i.v.* administration

Parameters	1b
T_max_ (h)	10.04±4.80
C_max_ (μg/mL)	43.02±15.04
AUC_0-t_ (μg/mL*h)	900.08±297.87
AUC_0-∞_ (μg/mL*h)	999.15±272.92
MRT_0-t_ (h)	16.03±0.41
MRT_0-∞_ (h)	22.27±4.96
T_1/2z_ (h)	14.52±6.32
CL (mL/h/g)	0.09±0.02

**Table 3:** Main pharmacokinetic parameters of **C10** for the saline- and **1b**-treated groups

Parameters	Saline-treated Group	1b-treated Group
T_max_ (h)	0.05±0.00	0.05±0.00
C_max_ (ng/mL)	1305.40±179.41	1633.02±344.88
AUC_0-t_ (ng/mL*h)	284.16±26.43	406.02±77.15
AUC_0-∞_ (ng/mL*h)	293.66±27.97	414.10±80.89
MRT_0-t_ (h)	0.11±0.02	0.15±0.02
MRT_0-∞_ (h)	0.16±0.04	0.17±0.03
T_1/2z_ (h)	0.36±0.16	0.19±0.02
Vz (mL/g)	0.80±0.33	0.30±0.06
CL (mL/h/g)	1.54±0.16	1.12±0.22
