# Supplementary material for: Amide naphthotube as a novel supramolecular sequestration agent for tetracaine and decamethonium
Source: Theranostics. 2024 Aug 19;14(13):5219–34. doi: 10.7150/thno.93654 (PMC11388068; doi:10.7150/thno.93654)
Supplement: Supplementary file 1 — Supplementary figures, materials and methods, and tables. [file thnov14p5219s1.pdf]

Supplemental Material

Amide naphthotube as a novel supramolecular sequestration agent for tetracaine and decamethonium

Authors: Cheng-Da Zhao[1], Wei Cai[2], Wen-Jie Chen[1], Huan Yao[1], Song-Meng Wang[3], Yan-Long Ma[4], Li-Li Wang[1], Liu-Pan Yang[1]

Affiliations:

[1]School of Pharmaceutical Science and School of Basic Medical Sciences, Hengyang Medical School, University of South China, Hengyang, 421001, China.

[2]School of Pharmaceutical Sciences, Hunan University of Medicine, Huaihua, 418000, China.

[3]Department of Chemistry, Southern University of Science and Technology, Xueyuan Blvd 1088, Shenzhen, 518055, China

[4]School of Chemistry and Chemical Engineering and Guangdong Cosmetics Engineering & Technology Research Center, Guangdong Pharmaceutical University, Zhongshan 528458, China.

Corresponding authors: Wei Cai, Yan-Long Ma, Li-Li Wang or Liu-Pan Yang. Email: 20120941161@bucm.edu.cn (Wei Cai), mayanlong202106@gdpu.edu.cn (Yan-Long Ma), wangll@usc.edu.cn (Li-Li Wang), yanglp@usc.edu.cn(Liu-Pan Yang).

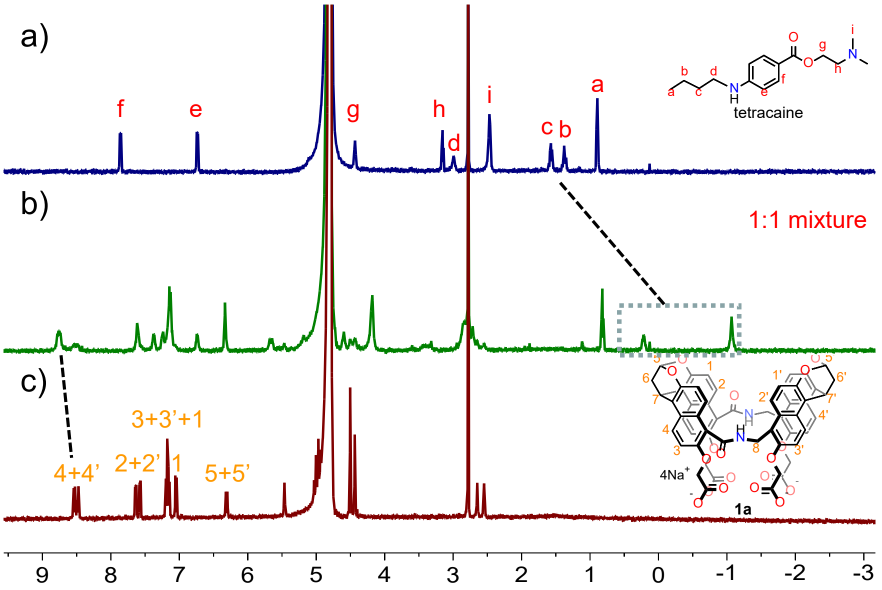

Figure S1. ^{1}H NMR spectra (500 MHz, 0.5 mM, 298 K) of (a) tetracaine, (c) **1a**, and (b) their equimolar mixture in PB buffer (pH =7.4). In the spectrum of their mixture, the aromatic protons of the host underwent obvious shifts, while the aromatic protons of the guest became broadened or disappeared into the baseline, supporting the binding between **1a** and tetracaine.

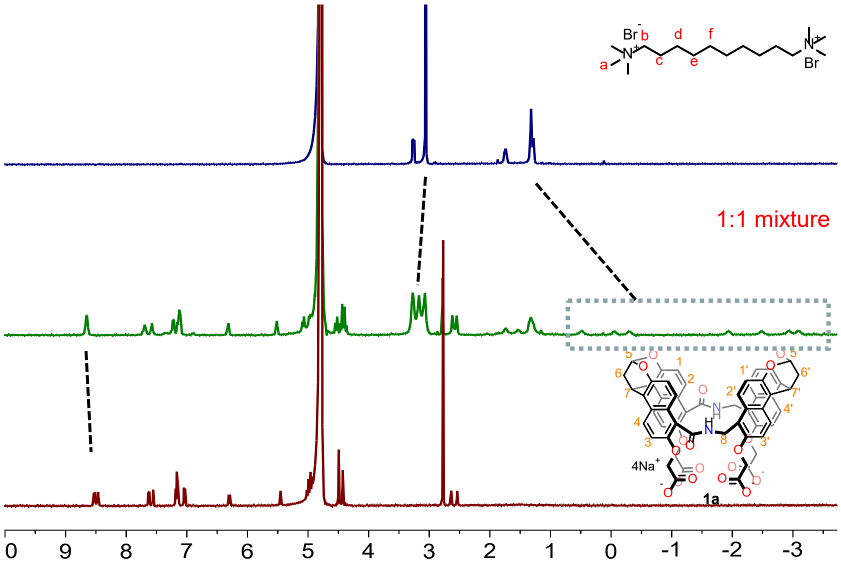

Figure S2. ^{1}H NMR spectra (500 MHz, 0.5 mM, 298 K) of (a) decamethonium, (c) **1a**, and (b) their equimolar mixture in PB buffer (pH =7.4). In the spectrum of their mixture, the signals of the guest become de-symmetrized obviously and the upfield shifts of the proton signals of the methylenes indicate the guest is within the cavity and experiences the shielding effect of aromatic rings.

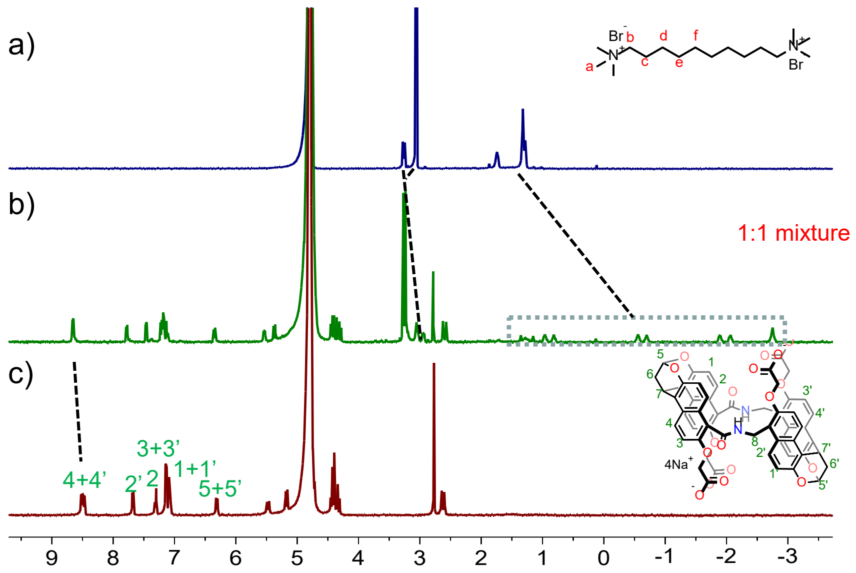

Figure S3. ^{1}H NMR spectra (500 MHz, 0.5 mM, 298 K) of (a) decamethonium, (c) **1a**, and (b) their equimolar mixture in PB buffer (pH =7.4). In the spectrum of their mixture, the signals of the guest become de-symmetrized obviously and the upfield shifts of the proton signals of the methylenes indicate the guest is within the cavity and experiences the shielding effect of aromatic rings.

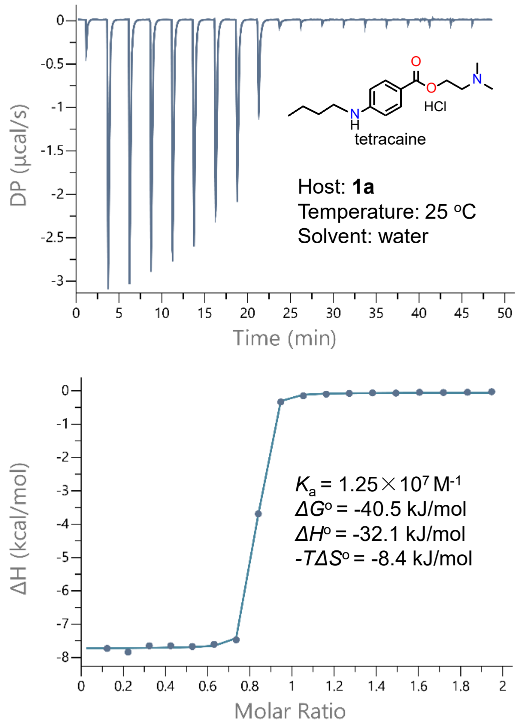

	K_a (M^{-1})	$\Delta G°$ (kJ/mol)
**1st**	1.25×10^7	-40.5
**2nd**	1.40×10^7	-40.8
**average**	1.32 ± 0.10)×10^7	-40.7

	$\Delta H°$ (kJ/mol)	$-T\Delta S°$ (kJ/mol)
**1st**	-32.1	-8.4
**2nd**	-33.7	-7.1
**average**	-32.9	-7.7

Figure S4. ITC titration plots (heat rate versus time and heat versus guest/host ratio) of tetracaine to **1a** in water at 25 °C.

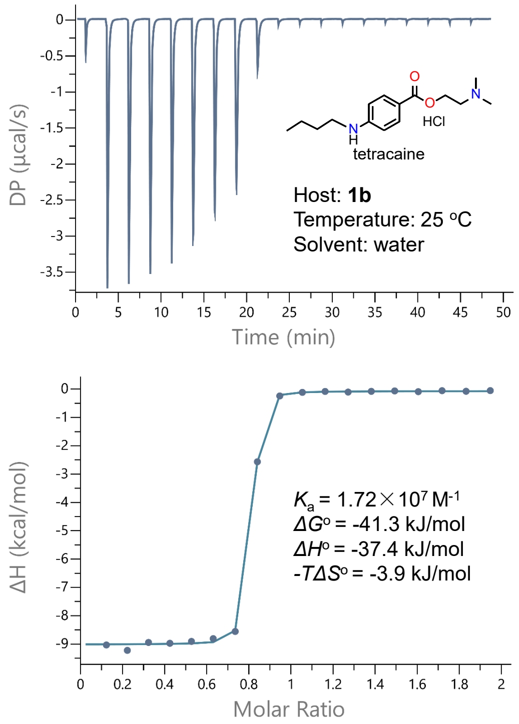

	K_a (M^{-1})	ΔG^o (kJ/mol)
**1st**	1.72×10^7	-41.3
**2nd**	2.07×10^7	-41.8
**average**	1.32 ± 0.10)×10^7	-40.7

	ΔH^o (kJ/mol)	$-T\Delta S^o$ (kJ/mol)
**1st**	-37.4	-3.9
**2nd**	-36.8	-4.9
**average**	-37.1	-4.4

Figure S5. ITC titration plots (heat rate versus time and heat versus guest/host ratio) of tetracaine to **1b** in water at 25 °C.

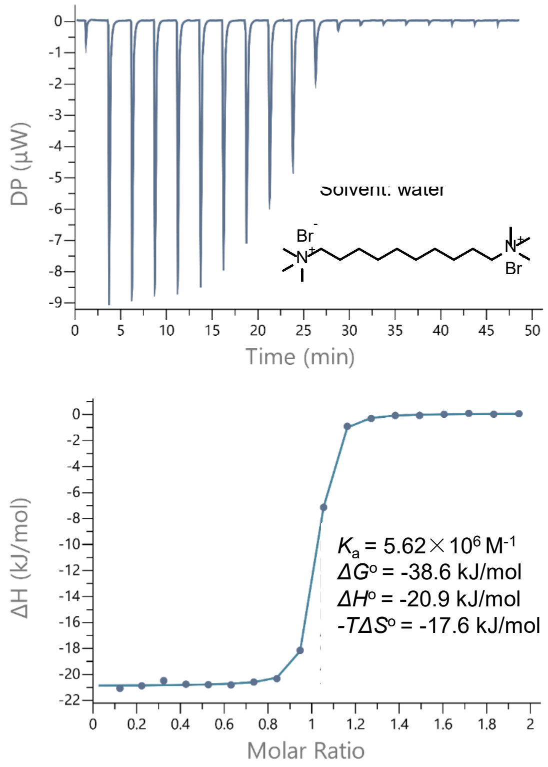

	K_a (M^{-1})	ΔG^o (kJ/mol)
**1st**	5.62×10^6	-38.6
**2nd**	5.85×10^6	-38.7
**average**	(5.73±0.1)×10^6	-38.6

	ΔH^o (kJ/mol)	$-T\Delta S^o$ (kJ/mol)
**1st**	-20.9	-17.6
**2nd**	-21.1	-17.6
**average**	-21.0	-17.6

Figure S6. ITC titration plots (heat rate versus time and heat versus guest/host ratio) of decamethonium to **1a** in water at 25 °C.

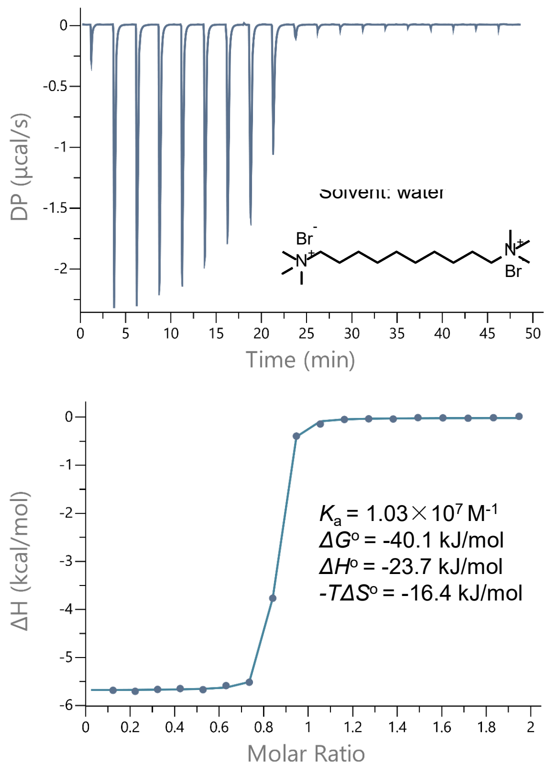

	K_a (M^{-1})	ΔG^o (kJ/mol)
**1st**	1.03×10^7	-40.1
**2nd**	1.00×10^7	-40.0
**average**	$(1.01\pm0.02)\times10^7$	-40.0

	ΔH^o (kJ/mol)	$-T\Delta S^o$ (kJ/mol)
**1st**	-23.7	-16.4
**2nd**	-24.2	-15.8
**average**	-23.9	-16.1

Figure S7. ITC titration plots (heat rate versus time and heat versus guest/host ratio) of decamethonium to **1b** in water at 25 °C.

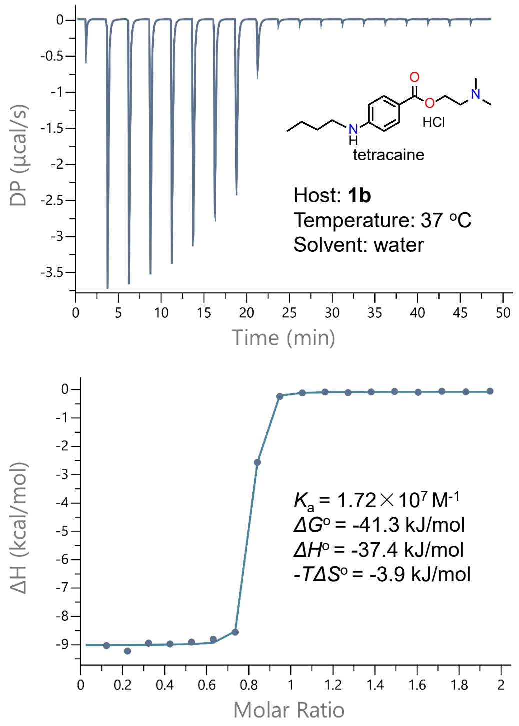

	K_a (M^{-1})	ΔG^o (kJ/mol)
**1st**	1.42×10^7	-42.4
**2nd**	1.37×10^7	-42.4
**average**	$1.40\pm0.04)\times10^7$	-42.4

	ΔH^o (kJ/mol)	$-T\Delta S^o$ (kJ/mol)
**1st**	-39.1	-3.3
**2nd**	-41.2	-1.2
**average**	-40.2	-2.2

Figure S8. ITC titration plots (heat rate versus time and heat versus guest/host ratio) of tetracaine to **1b** in water at 37 °C.

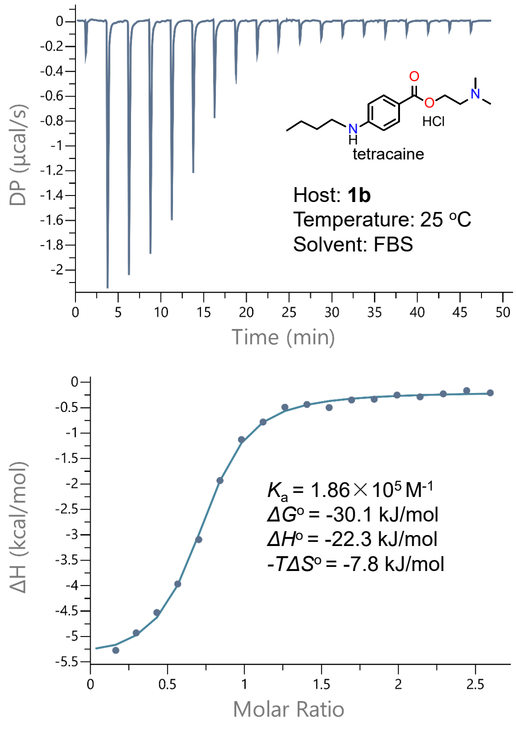

	K_a (M^{-1})	ΔG^{o} (kJ/mol)
**1st**	1.86×10^{5}	-30.1
**2nd**	1.61×10^{5}	-29.8
**average**	$1.74\pm0.17)\times10^{7}$	-29.9

	ΔH^{o} (kJ/mol)	$-T\Delta S^{o}$ (kJ/mol)
**1st**	-22.3	-7.8
**2nd**	-24.0	-5.7
**average**	-23.1	-6.8

Figure S9. ITC titration plots (heat rate versus time and heat versus guest/host ratio) of tetracaine to **1b** in FBS at 25 °C.

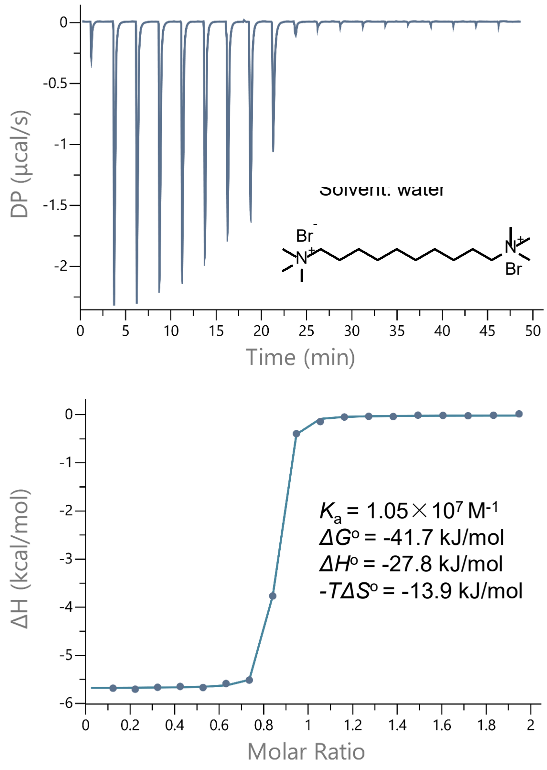

	K_a (M^{-1})	ΔG^{o} (kJ/mol)
**1st**	1.05×10^{7}	-41.7
**2nd**	8.62×10^{6}	-41.2
**average**	$(9.56\pm1.33)\times10^{6}$	-41.4

	ΔH^{o} (kJ/mol)	$-T\Delta S^{o}$ (kJ/mol)
**1st**	-27.8	-13.9
**2nd**	-28.4	-12.8
**average**	-28.1	-13.3

Figure S10. ITC titration plots (heat rate versus time and heat versus guest/host ratio) of decamethonium to **1b** in water at 37 °C.

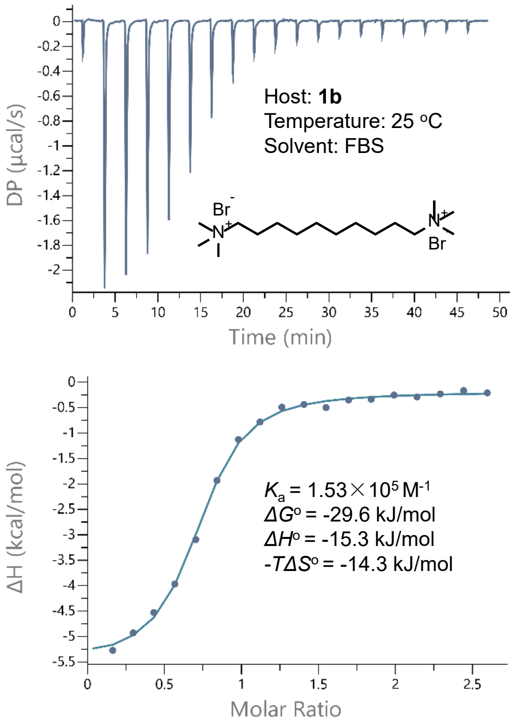

	K_a (M^{-1})	ΔG^o (kJ/mol)
**1st**	1.53×10^5	-29.6
**2nd**	1.36×10^5	-29.3
**average**	$(1.74 \pm 0.17)\times10^7$	-29.4

	ΔH^o (kJ/mol)	$-T\Delta S^o$ (kJ/mol)
**1st**	-15.3	-14.3
**2nd**	-15.3	-14.0
**average**	-15.3	-14.1

Figure S11. ITC titration plots (heat rate versus time and heat versus guest/host ratio) of decamethonium to **1b** in FBS at 25 °C.

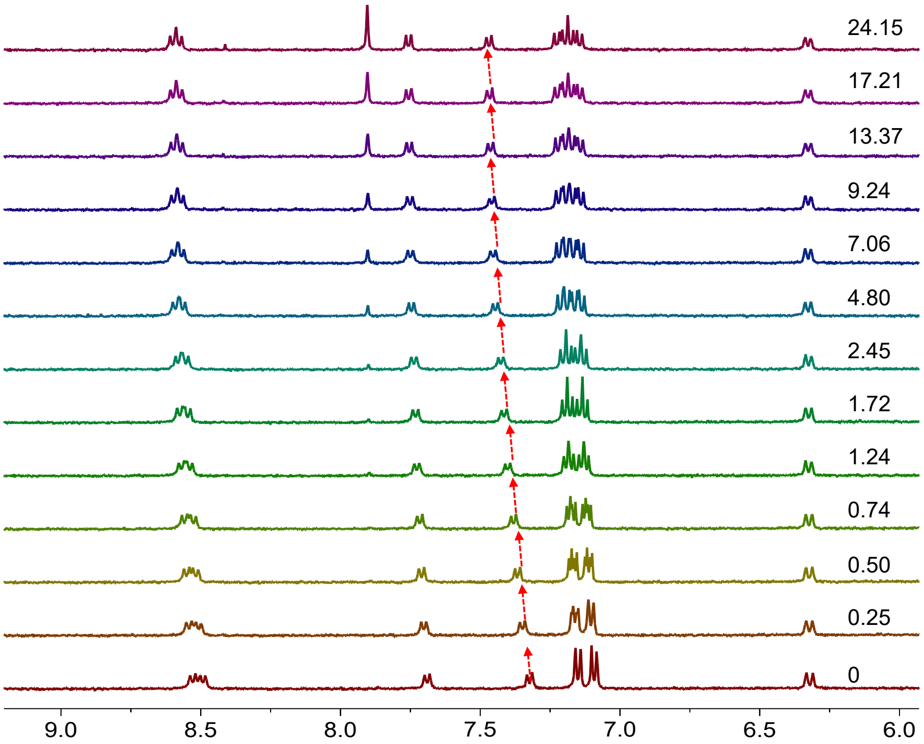

Figure S12. Partial ^{1}H NMR spectra (500 MHz, D_2O, 298 K) of **1b** (0.2 mM) titrated by **Acetylcholine**. From bottom to top, the concentration of **Acetylcholine** was 0~24.15 mM.

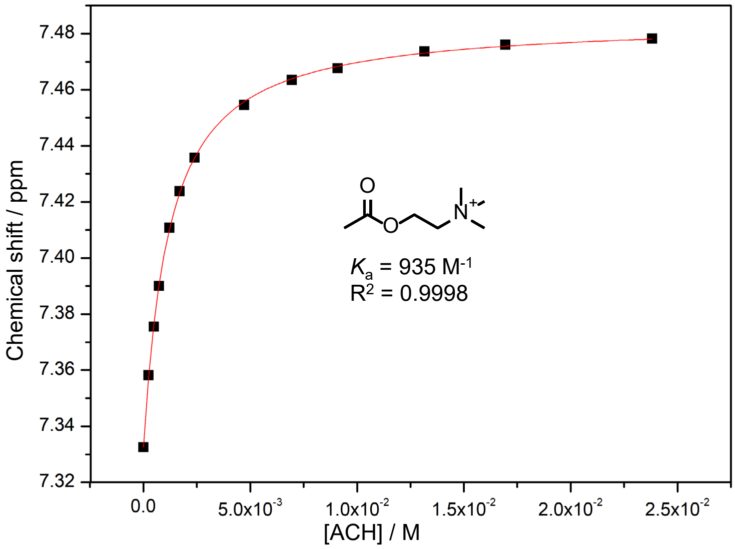

	Titration 1	Titration 2	average	error
K_1 (M^{-1})	1149	935	1042	151
R^2	0.9935	0.9998		

Figure S13. Non-linear curve-fitting for the complexation between **1b** and **Acetylcholine** in D_2O at 298 K.

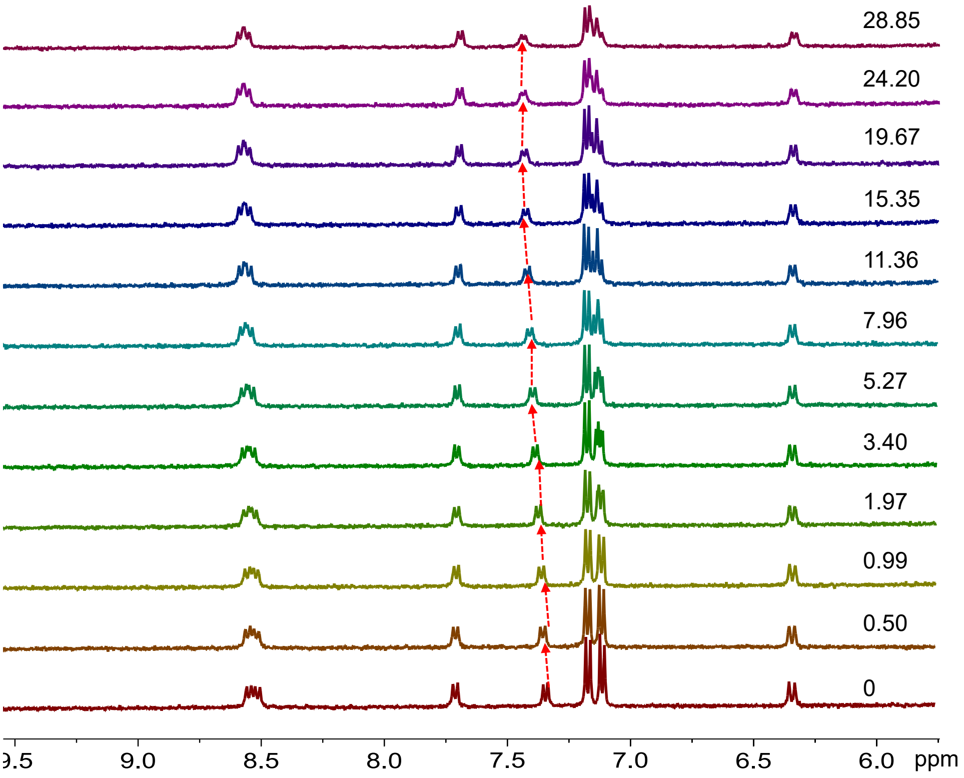

Figure S14. Partial ^{1}H NMR spectra (500 MHz, D_2O, 298 K) of **1b** (0.2 mM) titrated by **Choline chloride**. From bottom to top, the concentration of **Choline chloride** was 0~28.85 mM.

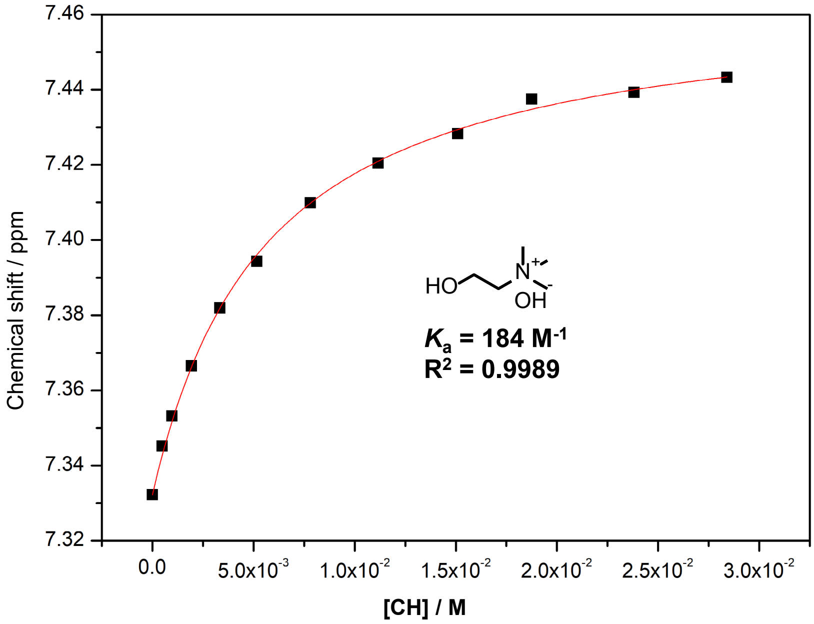

	Titration 1	Titration 2	average	error
K_1 (M^{-1})	155	184	169	21
R^2	0.9959	0.9989		

Figure S15. Non-linear curve-fitting for the complexation between **1b** and **Choline chloride** in D_2O at 298 K.

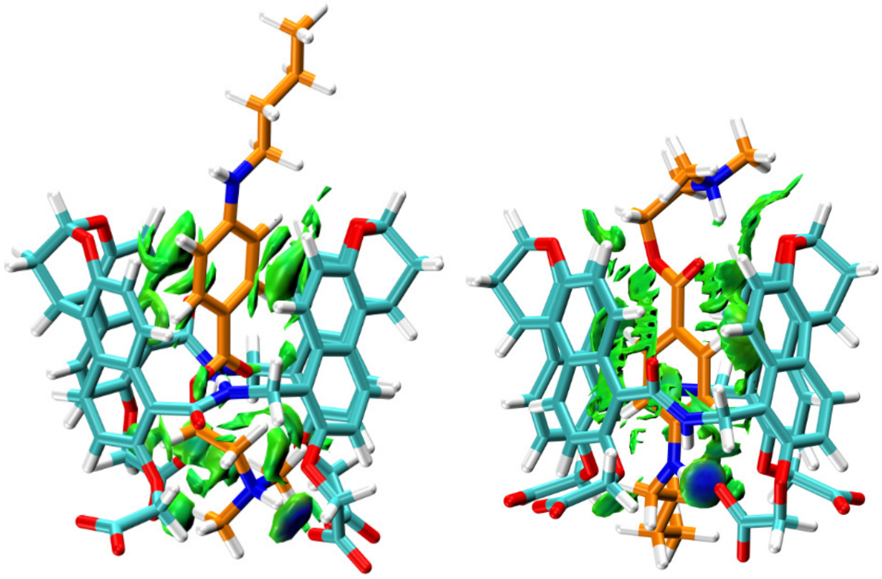

tetracaine@**1a**-conformer 1 -42.58 kJ/mol

tetracaine@**1a**-conformer 2 0 kJ/mol

Figure S16. Energy-minimized structures of two representative conformers of **TC@1a** using the wB97XD/def2-SVP method (ma-def2-SVP basis set was used for anionic parts) in water (PCM). The results show that **TC@1a**-conformer 1 is more energetically stable than **TC@1a**-conformer 2.

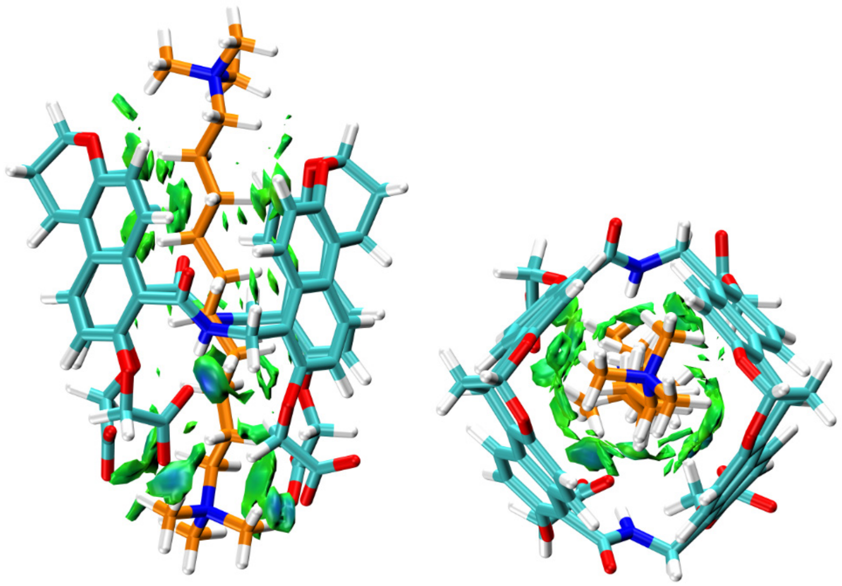

decamethonium@**1a**

Figure S17. Energy-minimized structures of **C10@1a** obtained by DFT calculations using wB97XD/def2-SVP method (ma-def2-SVP basis set was used for anionic parts) in water (PCM).

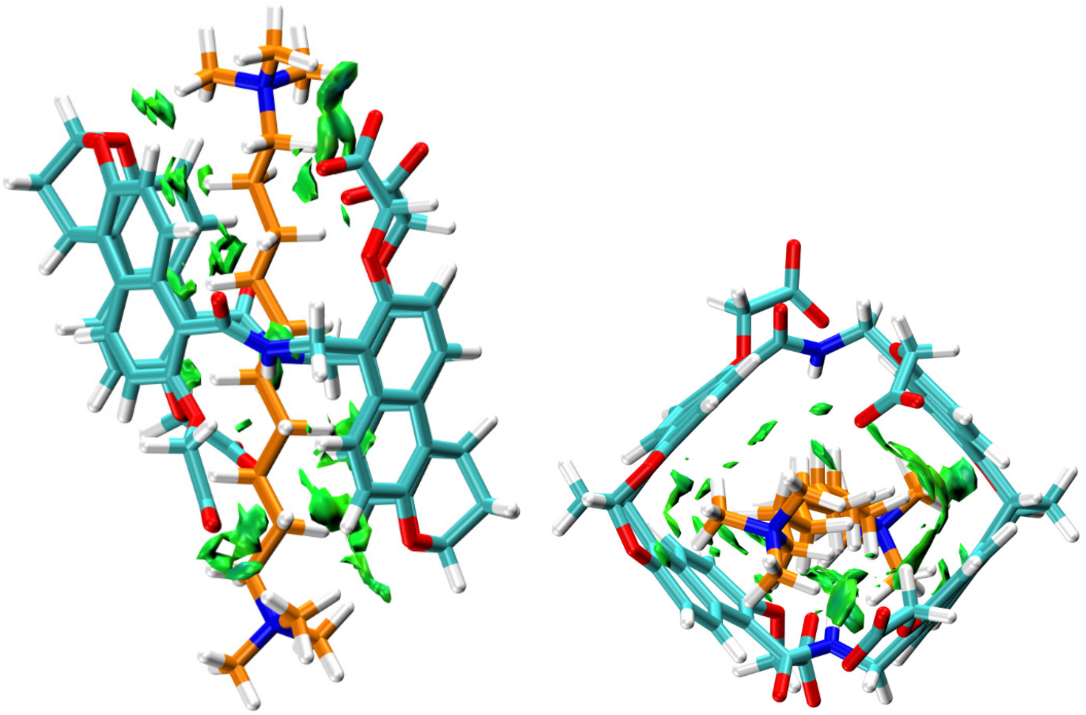

decamethonium@**1b**

Figure S18. Energy-minimized structures of **C10@1b** obtained by DFT calculations using wB97XD/def2-SVP method (ma-def2-SVP basis set was used for anionic parts) in water (PCM).

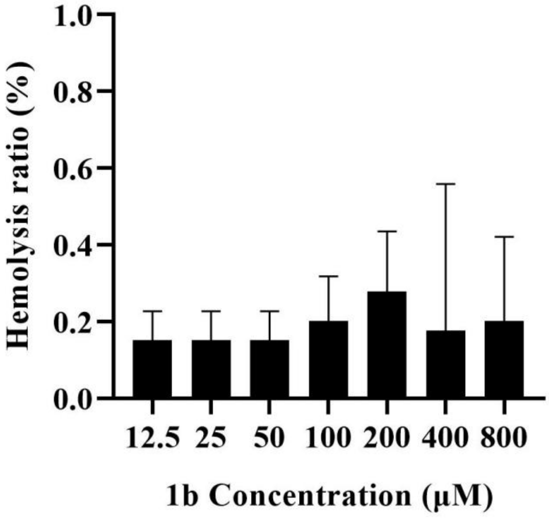

Figure S19. Hemolysis assay of **1b**. Data are presented as mean ± SD (n = 3)

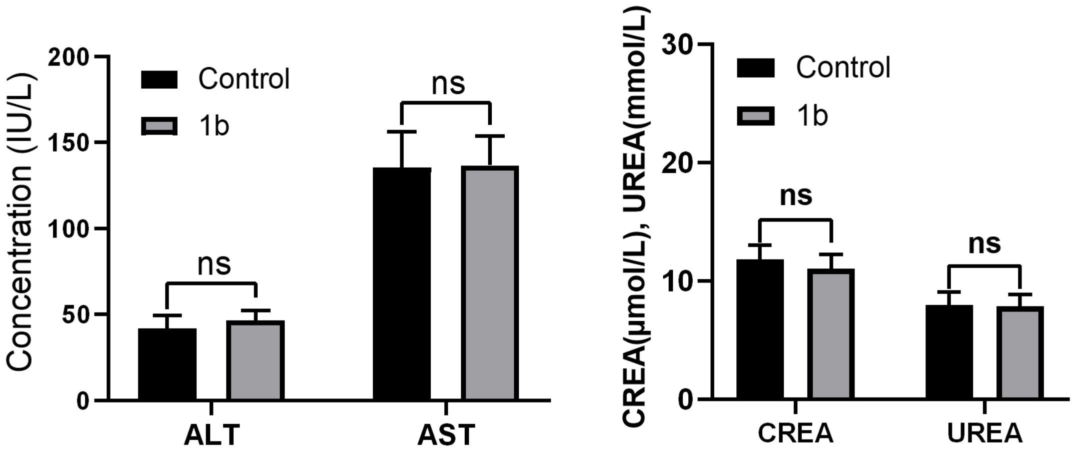

Figure S20. Hepatic (lef) and renal (B) function markers test on the blood samples collected from the mice on Day 1 after i.v. administration of 1b.

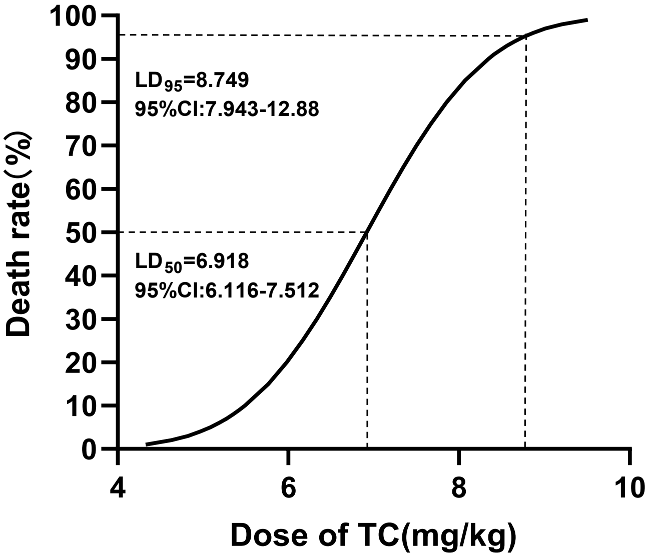

Figure S21. The dose-mortality curve of **TC** from probit analysis

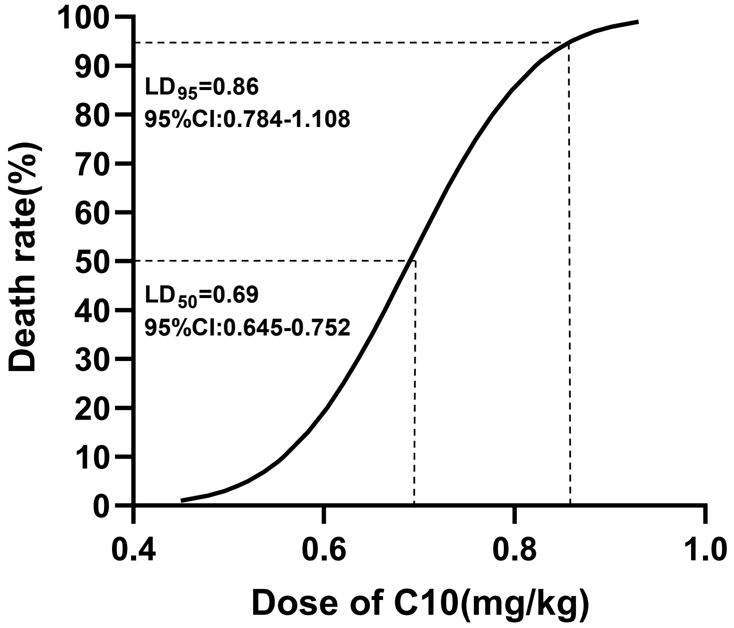

Figure S22. The dose- mortality curve of **C10** from probit analysis

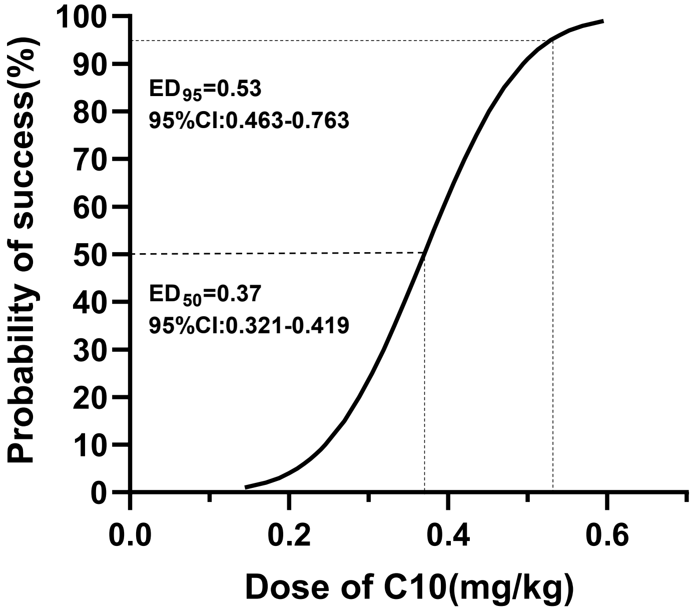

Figure S23. The dose-response curve of **C10** from probit analysis

Pharmacokinetics Experiments

1. Materials and Methods

1.1 Chemicals and Reagents

Lidocaine hydrochloride used as an internal standard (**IS**) was obtained from Aladdin. The purity of **C10**, **IS** and **1b** are all above 98.0%. LC-MS grade acetonitrile and methanol were supplied by Merck KGaA (Germany). Formic acid was procured from Thermo Fisher Scientific Ltd (USA). All other chemicals and reagents required for the experiment were of analytical grade.

1.2 LC/MS analysis

The LC/MS analysis was carried out by Liquid chromatography-mass spectrometry (Waters ACQUITY UPLC/Xevo TQ-S). Separation was performed on a Waters ACQUITY UPLC HSS T3 column (2.1 × 100 mm, 1.7 μm), using 0.1 % (v/v) formic acid in water (A) and acetonitrile (B) as mobile phase. The auto-sampler was kept at 10 ℃ and the injection volume was 2 μL. An optimized gradient program was established as follows: 5% B at 0-0.5 min, 5-95 % B at 0.5-4.0 min, 95-5 % B at 4.0-4.1 min, and 5 % B at 4.1-7 min. The flow rate was 0.3 mL/min, and the column temperature was set at 40 ℃. The parameters of the ESI source were set as follows: Capillary voltages, 1.0 kV; desolvation temp, 500 ℃; desolvation gas flow, 1000 L/Hr; Cone gas flow, 50 L/Hr; The analyte confirmation was performed by using retention time and multi-reaction monitoring (MRM) in positive ionization modes according to the optimized condition of each analyte (Table 1).

Table S1 Summary of target analytes and corresponding MRM parameters.

Analytes	tR(min)	Precursor Ions (m/z)	Product Ions (m/z)	Cone voltage （DP） （V）	Collision energy （CE） （eV）
**1b**	4.20	1055.5	497.5	60	42
**C10**	2.76	129	60	60	16
**IS**	3.26	234.9	85.9	45	18

1.3. Administration and collection of plasma samples

Male Kunming mice (30±2g) were randomly divided into 5 groups, namely **1b** group (dose 80mg/kg, concentration 20mg/ml), **C10** group (dose 0.45mg/kg, concentration 0.125mg/ml, **C10** injection for 30s followed by the same volume of normal saline), **1b** combined with **C10** group (**C10** dose 0.45 mg/kg, **C10** concentration 0.125mg/ml, C10 injection for 30s followed by **1b** 6 mg/kg, **1b** concentration 1.5 mg/ml). Drugs were injected through the tail vein of mice. In group **1b**, eyeball blood and liver and kidney tissues were collected at 0.25, 0.5, 1,1.5, 2, 4, 6, 8, 12, 24, 36 and 48 hours after administration, with 6 mice in each time point. In the other four groups, blood and liver and kidney tissues were collected from eyeballs at 3min, 5min, 10min, 15min, 30min, 45min and 1h after administration. Six mice were selected at each time point. Blood samples were placed in heparin sodium tubes and centrifuged at 6000rpm for 20 minutes at 4℃ to obtain plasma samples. They were stored in a refrigerator at -80 ° C and frozen until analysis. Plasma, liver and kidney tissues were stored in a freezer at -80 ℃ and then frozen until analysis was performed.

1.4. Preparation of Calibration Standards and Quality Control (QC) Samples

1b, C10, and **IS** were dissolved separately in methanol to get a stock solution concentration of 1.0 mg/mL. The stock solution of the drug to be tested was then diluted with methanol to prepare the working solution. Calibration standards and QC samples were prepared by diluting the working solution with methanol. The final concentrations of **C10** were 25, 250 and 1000 ng/mL, **1b** final concentration of 150, 1500 and 6000 ng/mL. The final concentration of **IS** is 10 ng/mL.

1.5. Mouse plasma sample preparation

The plasma to be tested was thawed at room temperature, and 100 μL of mouse plasma samples were collected in 1.5 mL EP tubes. 10 μL of IS solution (10 ng/mL) and 10 μL of methanol were mixed followed by the addition of 400 μL ethyl methanol-acetonitrile (1:1 v / v), and then the tubes were vortex mixed for 3.0 min. After centrifugation at 13,000 rpm for 20 min at 4 °C, the supernatant was transferred to a nitrogen blower, and the solvent was dried by maintaining a steady flow of nitrogen. After drying the solvent, reconstituted with 100 μL 20% aqueous methanol

solution, vortexed for 1 min, and centrifuged at 13000 rpm for 20 min, 2 μL of the supernatant was used for analysis in LC/MS.

1.6. Mouse liver and kidney sample preparation

Accurately weigh 200 mg of mouse liver or kidney samples, add 800 μL saline to the tissue grinding machine, centrifuge (4000 rpm / min, 4°C, 20min) with 200 μl supernatant, add 600 μl of methanol-acetonitrile (1:1 v / v) as protein precipitant, precision add 10 μL methanol solution (or QC solution) and 10 μL IS solution. The later experimental procedures refer to mouse plasma sample preparation.

1.7. Method Validation

This study was carried out in accordance with the 2020 Chinese Pharmacopoeia Part IV: Guidelines for Verification of Quantitative analytical methods for biological samples and the 2018 FDA Guidelines for Validation of biological sample analytical methods, etc. Low, medium and high quality control samples were selected for methodological investigation. The survey included the specificity of the analysis method, linearity, precision and accuracy, extraction recovery, and matrix effects.

1.7.1 Specificity

Specificity was determined by comparison with blank plasma samples and chromatograms of blank plasma samples supplemented with **C10**, **1b**, and **IS** and plasma samples obtained after intravenous injection.

1.7.2 Standard curve and the linear range

The concentration of each compound in plasma was taken as the abscissa (X), the ratio of the peak area of the compound to the peak area of IS was taken as the ordinate (Y), and the weighted (W=1 / X) least squares linear regression was used to obtain the regression equation of each compound.

1.7.3 Precision and Accuracy

Precision and accuracy were obtained by analyzing QC samples six times in duplicate on the same day (intra-day) and on three consecutive days (inter-day) at three concentration levels (low, medium, and high concentration). Relative standard deviation (RSD) was used to evaluate precision, and relative error (RE) was used to evaluate accuracy.

1.7.4 Extraction Recovery and Matrix Effect

A total of 100μL of blank mouse plasma was previously removed, and QC sample solutions of low, medium and high concentrations were added, respectively, with 6 aliquots of each concentration in parallel. According to the above plasma sample processing procedures and LC/MS analysis methods, the peak areas of each tested compound and internal standard were determined and recorded as A; (2) The blank rat plasma (100 μL) was treated by the plasma sample processing method mentioned above, and then QC sample solution of low, medium and high concentration was added to redissolve in 6 aliquots in parallel. The peak areas of each compound were determined by injection analysis and recorded as B. (3) The peak areas of each compound were directly determined in 6 parallel samples of QC samples with low, medium and high concentrations and recorded as C. The extraction recovery rate R=A/B×100%; Matrix effect E=B/C×100%.

1.8 Data handling

The Masslynx 4.1 software was used to obtain the peak area of the tested compounds and internal standards in each plasma sample and calculate the standard curve formula. Their plasma concentration data were processed using DAS 3.2.8 software and pharmacokinetic parameters were calculated.

2. Results

2.1. Specificity

By performing specialized analysis on blank mouse plasma, spiked mouse plasma, and actual mouse plasma samples, as depicted in Figure below, it was found that the blank mouse plasma did not contain target compounds. The retention times for **1b**, **C10**, and lidocaine were 4.20 min, 2.76 min, and 3.26 min, respectively. Each compound exhibited good peak shapes, and there were no interfering peaks observed in the blood samples.

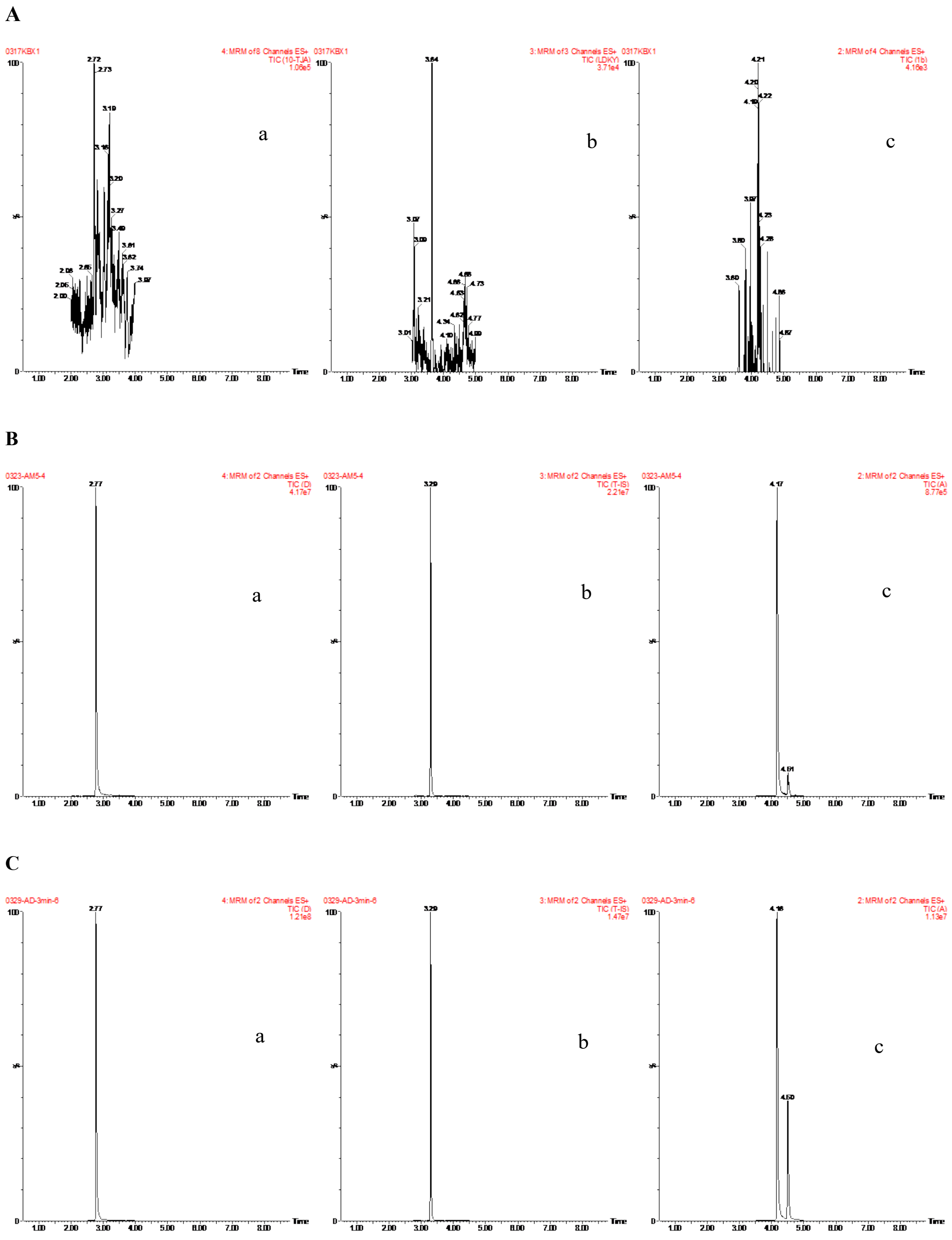

Figure S24 Specialized mass spectra of target compounds and internal standards: **(A)** Blank plasma; **(B)** Blank plasma spiked with QC and internal standards; **(C)** Post-dose mouse plasma; where a corresponds to **C10**, b corresponds to lidocaine, c corresponds to **1b**.

Table S2. Standard curves and linear ranges for **1b** and **C10** in mouse plasma

Compound	Standard curve	R^2	Linear Range (ng/mL)	Limit (ng/mL)
**1b**	Y=0.000569201X+0.009139	0.9941	20.58-80000	13.08
**C10**	Y=0.0596726X+0.246099	0.9955	2.06-8000	1.10

Table S3. Precision and accuracy data of **1b** and **C10** in mouse plasma (*n* = 6).

		Intra-day precision			Inter-day precision		
Compound	Concentration (ng/ml)	Measured value(ng/mL)	Accuracy (%)	RSD (%)	Measured value(ng/mL)	Accuracy (%)	RSD (%)
**C10**	25	25.72±0.73	103.70	2.85	26.83±1.03	107.30	3.82
	250	277.61±8.47	111.05	3.05	274.86±2.51	109.95	0.91
	1000	992.59±2.41	99.26	0.24	991.25±1.97	99.12	0.20
**1b**	150	125.09±15.62	83.39	12.49	121.00±4.55	80.67	3.76
	1500	1489.95±183.07	99.33	12.29	1517.55±85.55	101.17	5.66
	6000	5537.15±319.87	92.29	5.78	5262.70±280.12	87.71	5.32

Table **S4**. Recovery and matrix effect (%) data for the analytes in mouse plasma (*n* = 6)

Compound	Concentration (ng/ml)	Matrix Effect (%)	RSD%	Recovery **(%)**	RSD%
**C10**	25	99.06±6.97	7.04	100.41±7.78	7.75
	250	115.03±4.74	4.12	91.61±7.89	8.61
	1000	106.50±5.29	4.97	97.19±2.74	2.82
**1b**	150	87.36±3.81	4.36	85.20±4.00	4.69
	1500	92.00±5.74	6.24	84.79±8.63	10.17
	6000	87.95±4.38	4.98	79.62±6.56	8.24
Lidocaine(IS)	10	99.75±4.57	4.58	82.43±1.25	1.52

Table **S5**. Standard curves and linear ranges for **1b** in mouse liver and kidney

Standard curve	R^2	Linear Range (ng/ml)	Limit(ng/ml)
Y=0.000715107X+0.0325573	0.9922	3.43-20000	3.43
